# Structural and dynamic insights revealing how lipase binding domain MD1 of *Pseudomonas aeruginosa* foldase affects lipase activation

**DOI:** 10.1038/s41598-020-60093-4

**Published:** 2020-02-27

**Authors:** Aldino Viegas, Peter Dollinger, Neha Verma, Jakub Kubiak, Thibault Viennet, Claus A. M. Seidel, Holger Gohlke, Manuel Etzkorn, Filip Kovacic, Karl-Erich Jaeger

**Affiliations:** 1 Institute of Physical Biology, Heinrich Heine University Düsseldorf, 40225 Düsseldorf, Germany; 2 Institute of Molecular Enzyme Technology, Heinrich Heine University Düsseldorf, 52425 Jülich, Germany; 3 Institute for Pharmaceutical and Medicinal Chemistry, Heinrich Heine University Düsseldorf, 40225 Düsseldorf, Germany; 4 Institute of Biological Information Processing, IBI-7: Structural Biochemistry, Forschungszentrum Jülich GmbH, 52425 Jülich, Germany; 5 Institute of Molecular Physical Chemistry, Heinrich Heine University Düsseldorf, 40225 Düsseldorf, Germany; 6 John von Neumann Institute for Computing (NIC) and Jülich Supercomputing Centre (JSC), Forschungszentrum Jülich GmbH, 52425 Jülich, Germany; 7 JuStruct: Jülich Center for Structural Biology, Forschungszentrum Jülich GmbH, 52425 Jülich, Germany; 8 Institute of Bio- and Geosciences IBG-1: Biotechnology, Forschungszentrum Jülich GmbH, 52425 Jülich, Germany; 9 Present Address: UCIBIO, Departamento de Química, Faculdade de Ciências e Tecnologia, Universidade Nova de Lisboa, 2829-516 Caparica, Portugal

**Keywords:** Hydrolases, Biotechnology

## Abstract

Folding and cellular localization of many proteins of Gram-negative bacteria rely on a network of chaperones and secretion systems. Among them is the lipase-specific foldase Lif, a membrane-bound steric chaperone that tightly binds (*K*_D_ = 29 nM) and mediates folding of the lipase LipA, a virulence factor of the pathogenic bacterium *P. aeruginosa*. Lif consists of five-domains, including a mini domain MD1 essential for LipA folding. However, the molecular mechanism of Lif-assisted LipA folding remains elusive. Here, we show in *in vitro* experiments using a soluble form of Lif (*s*Lif) that isolated MD1 inhibits *s*Lif-assisted LipA activation. Furthermore, the ability to activate LipA is lost in the variant *s*Lif_Y99A_, in which the evolutionary conserved amino acid Y99 from helix α1 of MD1 is mutated to alanine. This coincides with an approximately three-fold reduced affinity of the variant to LipA together with increased flexibility of *s*Lif_Y99A_ in the complex as determined by polarization-resolved fluorescence spectroscopy. We have solved the NMR solution structures of *P. aeruginosa* MD1 and variant MD1_Y99A_ revealing a similar fold indicating that a structural modification is likely not the reason for the impaired activity of variant *s*Lif_Y99A_. Molecular dynamics simulations of the *s*Lif:LipA complex in connection with rigidity analyses suggest a long-range network of interactions spanning from Y99 of *s*Lif to the active site of LipA, which might be essential for LipA activation. These findings provide important details about the putative mechanism for LipA activation and point to a general mechanism of protein folding by multi-domain steric chaperones.

## Introduction

The Gram-negative human pathogen *Pseudomonas aeruginosa* produces a wide range of extracellular enzymes^1,2^, among them the lipase LipA, a secreted putative virulence factor^3–5^. For its conversion into an enzymatically active conformation, LipA requires the assistance of an inner membrane-bound chaperone named lipase-specific foldase (Lif)^6^. On the folding pathway, LipA can adopt several structurally different intermediates: an inactive and unfolded molten globule-like conformation^7^, a near-natively folded pre-active conformation^8^ and two folded conformations that differ in the structure of the α-helical lid covering the active site, with the folded closed conformation being enzymatically inactive and the folded open conformation enzymatically active^9,10^. Addition of Lif to the pre-active lipase immediately activates the folding intermediate^11–16^, suggesting that the interactions with Lif help overcoming an energetic barrier on the folding pathway of lipase LipA.

**Figure 1:**
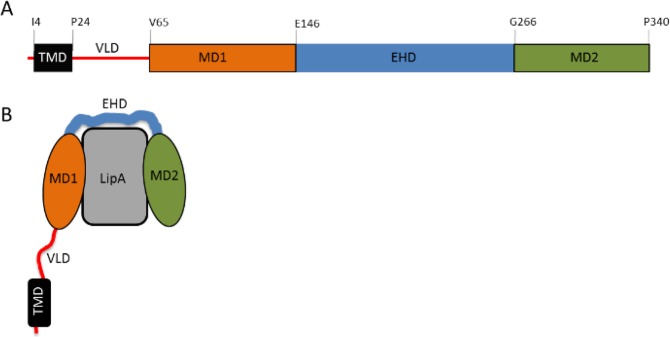
Schematic representation of *P. aeruginosa* Lif and its complex with lipase LipA. (**A**) Five-domain organization of Lif and (**B**) Lif-LipA complex. The catalytic folding domain (CFD) self-sufficient for activation of LipA *in vitro* comprises MD1, EHD and MD2. Residues defining the beginning and the end of each domain are indicated in (**A**).

Lif proteins constitute a unique class of steric chaperones^17,18^. *P. aeruginosa* Lif has a five-domain organization with a transmembrane α-helical domain (TMD), followed by a probably unstructured variable linker domain (VLD) and the catalytic folding domain (CFD) which interacts with the lipase (Fig. 1). The crystal structure of *Burkholderia glumae* foldase (homologous to *P. aeruginosa* foldase) in complex with its cognate lipase reveals only the periplasmic catalytic folding domain^10^. This domain consists of 11 α-helices connected by loops and is organized into two globular domains, mini-domain 1 (MD1, α1-α3) and mini-domain 2 (MD2, α9-α11), which are connected by the highly flexible extended helical domain (EHD, α4-α8). Six α-helices of Lif (α1, α4, α5, α7, α9, α11) are in direct contact with LipA, forming a large interface between Lif and LipA, which is consistent with the high binding affinity in the nanomolar range of these two molecules^10^.

The sequence alignment of *P. aeruginosa* foldase (*Pa*Lif) and *B. glumae* foldase (*Bg*Lif), the only foldase with known 3D structure, revealed that among the five domains MD1 shares the highest sequence similarity (52%) (Fig. S1). Similar sequences often exert similar functions, which holds for MD1, as the chimeric foldase of *B. glumae* containing the MD1 of *P. aeruginosa* Lif still activated *B. glumae* LipA^8^. In contrast, other hybrid *B. glumae*-*P. aeruginosa* Lifs with replaced MD2 and EHD were inactive and *B. glumae* Lif did neither activate *P. aeruginosa* LipA nor *vice versa*^8^. The importance of MD1 for foldase activity was further highlighted by the finding that MD1 comprises the foldase sequence motif RXXFDY(F/C)L(S/T)A (X can be any residue) which is evolutionarily strongly conserved among all foldase families^12^ and which when mutated inactivates foldase^19^. However, despite this detailed knowledge, the molecular mechanism of foldase-assisted lipase folding still remains elusive.

Here, we investigated the role of MD1 for the activation of pre-active LipA by biochemical analysis, NMR spectroscopy, fluorescence spectroscopy and molecular simulations. Our solution NMR structures reveal that mutation Y99A in MD1 induces only slight changes in the protein structure. However, our biochemical activation assays show that in contrast to MD1, MD1_Y99A_ does not decelerate *s*Lif-induced activation of LipA. *s*Lif is a soluble form of *Pa*Lif that lacks the TMD and Y99 is evolutionary conserved and located in helix α1 of MD1. While sLif and *s*Lif_Y99A_ both interact with LipA, fluorescence-based assays demonstrate that the mutation significantly reduces the sLif-LipA affinity. The role of mutation Y99A on LipA activation was probed by molecular dynamics (MD) simulations and rigidity theory. Comparative constraint network analyses of MD-generated conformational ensembles of wild-type *s*Lif and variant *s*Lif_Y99A_ in complex with LipA suggest that long-range network interactions spanning from Y99 of *s*Lif to the active site of LipA are likely involved in LipA activation.

## Results

### Isolated MD1, but not MD1_Y99A_ decelerates sLif-induced activation of LipA

**Figure 2:**
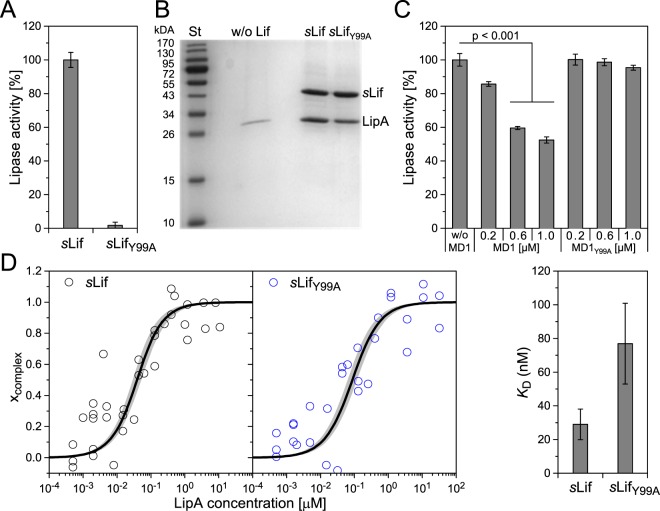
Activation of *P. aeruginosa* lipase LipA with *s*Lif and variant *s*Lif_Y99A_ and effect of MD1. (**A**) Pre-active LipA (4 µM) was incubated with either *s*Lif (4 µM) or variant *s*Lif_Y99A_ (4 µM) followed by lipase activity assay with 10 nM LipA. The activity of *s*Lif:LipA complex was set as 100%. (**B**) SDS-PAGE analysis of LipA co-purified in the complex with *s*Lif or *s*Lif_Y99A_ as well as without Lif (w/o Lif). In the Coomassie Brilliant Blue G250 stained gel LipA is migrating as ~30 kDa and *s*Lif as 43 kDa protein. Molecular weights of standard proteins (St) are indicated on the left-hand side. (**C**) Inhibition of *s*Lif-mediated LipA activation with MD1. Pre-active LipA (50 nM) incubated with MD1 or MD1_Y99A_ was activated by addition of *s*Lif (50 nM) and 10 min incubation prior to lipase activity measurement. Lipase activities are mean values ± standard deviation of three independent experiments each measured with at least three samples. (**D**) A fluorescence assay was used to study the complex formation of pre-active LipA and *s*Lif/*s*Lif_Y99A_ labelled at amino groups with BDP FL. The fraction of fluorescence parameters assigned to the *s*Lif:LipA complex (steady-state anisotropy *r*_steady-state_ (Eq. 2, Table S1) and average translational diffusion time ‹*t*_trans_› (Eq. 3, Table S2). The binding data were fitted with a 1:1 binding affinity model (Eq. 4), black line. The uncertainties are indicated as shaded areas. Steady-state anisotropy could not be used for *s*Lif_Y99A_:LipA complex because increased mobility of the fluorescent probe cancels the increase of global rotation correlation time ρ_global_. The apparent dissociation constant *K*_D_ (right panel) was determined to 29 nM ± 9 nM for *s*Lif:LipA and 77 nM ± 24 nM for *s*Lif_Y99A_:LipA complex (error bars are standard errors of the fit).

In line with previous results^19^ we observed that the point mutation generating variant *s*Lif_Y99A_ strongly modifies *in vitro* folding of LipA (*s*Lif lacks the TMD, which is dispensable for *in vitro* Lif function^14^ (Fig. 2A). Interestingly, however, LipA strongly binds to both *s*Lif and variant *s*Lif_Y99A_ (Fig. 2B). Presumably, specific interactions of MD1 with LipA are important for its activation as proposed for activation of *B. glumae* lipase, too^8,10,19^. We purified MD1 (Fig. S2) and demonstrated that these interactions are not sufficient for LipA activation as isolated MD1 could not activate pre-active LipA *in vitro* (data not shown). However, the addition of MD1 to pre-active LipA in 12 to 20-fold molar excess during activation of LipA with *s*Lif significantly (p < 0.001, n = 4) slowed down the activation reaction (Fig. 2C). This result indicates that isolated MD1 can interfere with *s*Lif’s capability to activate LipA. This effect was not observed with isolated MD1_Y99A_ (Fig. 2C). We analyzed the affinity of both *s*Lif and variant *s*Lif_Y99A_ to LipA using a fluorescence-based assay (Tables S1 and S2). We observed stronger binding of *s*Lif (*K*_D_ = 29 ± 9 nM) than *s*Lif_Y99A_ (*K*_D_ = 77 ± 24 nM) (Fig. 2D).

### LipA complexes with sLif and variant sLif_Y99A_ exhibit similar unfolding profiles

**Figure 3:**
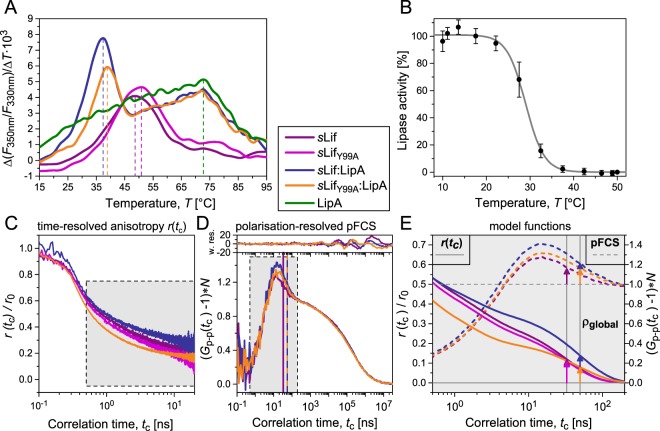
Effect of temperature on unfolding and activity of LipA in complex with *s*Lif and variant *s*Lif_Y99A_. (**A**) Melting curves of *s*Lif, *s*Lif_Y99A_, pre-active LipA alone and after incubation with *s*Lif, *s*Lif_Y99A_, MD1 and MD1_Y99A_ obtained by fluorescence measurement with nanoDSF. (**B**) Temperature-dependent lipase activity in a solution of sLif and LipA generated by incubation of pre-active LipA (100 nM) with *s*Lif (250 nM) overnight at 4 °C in TG buffer. Samples were then incubated at different temperatures (10–50 °C) for 1 h followed by measurement of the remaining lipase activity with 2 nM LipA. (**C**) Time-resolved fluorescence anisotropy decay curves *r*(*t*_c_) of free and complex sLif and *s*Lif_Y99A_. Open circles indicate experimental *r*(*t*_c_) and lines indicate model *r*(*t*_c_) (Eq. S2b, results see Table S4), dashed lines indicate complex. Further details see main text. (**D**) Polarization-resolved full-FCS of labelled *s*Lif using the p-p cross-correlation curves *G*_p-p_(*t*_c_) normalised to the number of molecules in focal volume (Eq. S3) together with weighted residuals of the fits (upper plot, Eq. S4, results in Table S5). The global rotation correlation time ρ_global_ of *s*Lif is similar to the one obtained by anisotropy measurement (32 ± 3 ns, indicated by vertical line). The global rotation correlation times of *s*Lif:LipA and *s*Lif_Y99A_:LipA are similar (50 ± 3 ns, vertical line). (**E**) Joint analysis of the anisotropy order parameters (solid lines, see shaded area in **C**) and normalized pFCS amplitudes (dashed lines, see shaded area in **D**) by displaying the model functions of the fits. The global rotational correlation times are depicted as vertical lines. The corresponding amplitudes are highlighted by arrows. For further details see main text.

We further probed the interactions of MD1, MD1_Y99A_, *s*Lif and *s*Lif_Y99A_ with pre-active LipA by analyzing the intrinsic protein fluorescence of respective solutions during thermal unfolding using a Prometheus nanoDSF device. While *s*Lif and *s*Lif_Y99A_ alone show typical unfolding curves with unfolding temperatures of ~50 °C (Fig. 3A), the thermal unfolding of MD1 and MD1_Y99A_ cannot be monitored with nanoDSF because MD1 does not contain tryptophan residues. The thermal unfolding curve of pre-active LipA does not show a sharp unfolding transition typical for folded proteins but rather a broad transition with a maximum at 73 °C. Addition of MD1 or MD1_Y99A_ to pre-active LipA did not considerably affect this unfolding curve, indicating that the domains do not change the fold of pre-active LipA and/or do not strongly interact with LipA (Fig. S3), which was also confirmed by fluorescence binding assay (Figs. S4 and S5 and Table S3).

In contrast, the addition of *s*Lif or *s*Lif_Y99A_ to pre-active LipA yielded typical unfolding curves with a maximum of the first derivatives at ~37 °C (Fig. 3A). Further heating of the solutions above 37 °C resulted in unfolding curves similar to the one observed for pre-active LipA (Fig. 3A). The unfolding temperatures for *s*Lif or *s*Lif_Y99A_ in the presence of LipA are ~7 °C higher than the temperature at which LipA activity is reduced to 50% in a temperature-dependent lipase activity assay in the presence of *s*Lif (half-inactivation temperature, *T*_50_ = 29.0 ± 0.2 °C, Fig. 3B); the temperature difference may be explained by the perturbation of the contacts within *s*Lif:LipA complex.

### Internal flexibility of variant sLif_Y99A_ in complex with LipA is increased as compared to sLif

Rotational diffusion and flexibility of *s*Lif were investigated by different techniques of polarization-resolved fluorescence spectroscopy^20^. The fluorescence anisotropy decay *r*(*t*_c_) of the fluorescent probe, Bodipy FL NHS ester (BDP FL) conjugated to *s*Lif or *s*Lif_Y99A_, is sensitive to local flexibility (time scale of 0.1 to 10 ns) and global rotation of the labelled molecules (Figs. 3C and S6). Typical fluorescence lifetime data and depolarization times are compiled in Table S4. Polarization-resolved full-FCS (pFCS) is sensitive to depolarization motions in a time range >10 ns (Fig. 3D), so that a joint analysis of time-resolved anisotropy and pFCS can capture a much wider time range (0.1 ns to ms) (Fig. 3E).

Considering free sLif and sLif_Y99A_, the fluorescence anisotropy (Fig. 3C) and pFCS (Fig. 3D) show that *s*Lif and *s*Lif_Y99A_ exhibit similar hydrodynamic properties (rotational correlation time global ρ_global_ = 32 ± 3 ns). Similar high amplitudes of pico- to nanosecond dynamics are evidence for a high degree of internal flexibility of the protein in the absence of LipA (order parameter, *S*^2^ = 0.30, Fig. 3C, Table S4) that usually results in dynamic conformational ensemble^21^, which agrees with the findings of Pauwels *et al*.^7^.

For the complexes *s*Lif:LipA and *s*Lif_Y99A_:LipA the global rotational correlation time is nearly doubled (ρ_global_ = 50 ± 3 ns). This experimental value agrees with the structure-based theoretical value for the global rotational correlation time ρ_global_ = 45 ns obtained by HydroPRO^22^ using the crystal structure of the complex of *B. glumae* PDB code (2ES4)^10^. The fact that ρ_global_ in free *s*Lif is reduced indicates that its conformation must be more collapsed. The comparison of order parameters in Fig. 3E gives further insights into the internal interactions. Notably, the comparison shows that the two independent fluorescence techniques agree very well with respect to observed depolarization times and amplitudes.

In the case of the *s*Lif:LipA complex, the increased order parameter *S*^2^ = 0.38 for global motion (blue arrow in Fig. 3E) indicates that *s*Lif is less flexible in its bound form. However, for *s*Lif_Y99A_:LipA, the order parameter for global motion (*S*^2^ = 0.22, orange arrow) is even further decreased as compared to free *s*Lif_Y99A_ (*S*^2^ = 0.30, Fig. 3E). This finding agrees well with the result that *s*Lif_Y99A_:LipA forms a less tight complex (Fig. 2D) as compared to wt *s*Lif. At the same time, the complex formation of *s*Lif_Y99A_ with LipA disrupts many internal interactions in free *s*Lif_Y99A_ so that internal friction should be reduced due to missing contacts. To conclude, the number of contacts in the *s*Lif_Y99A_:LipA complex is significantly reduced. As the Y99A mutation is in MD1 domain, we can further conclude that for the mutant the MD1 domain does not, or only weakly, interact with LipA while the rest of *s*Lif should still interact normally.

### Structural insights into MD1s of *P. aeruginosas*Lif and variant *s*Lif_Y99A_

**Figure 4:**
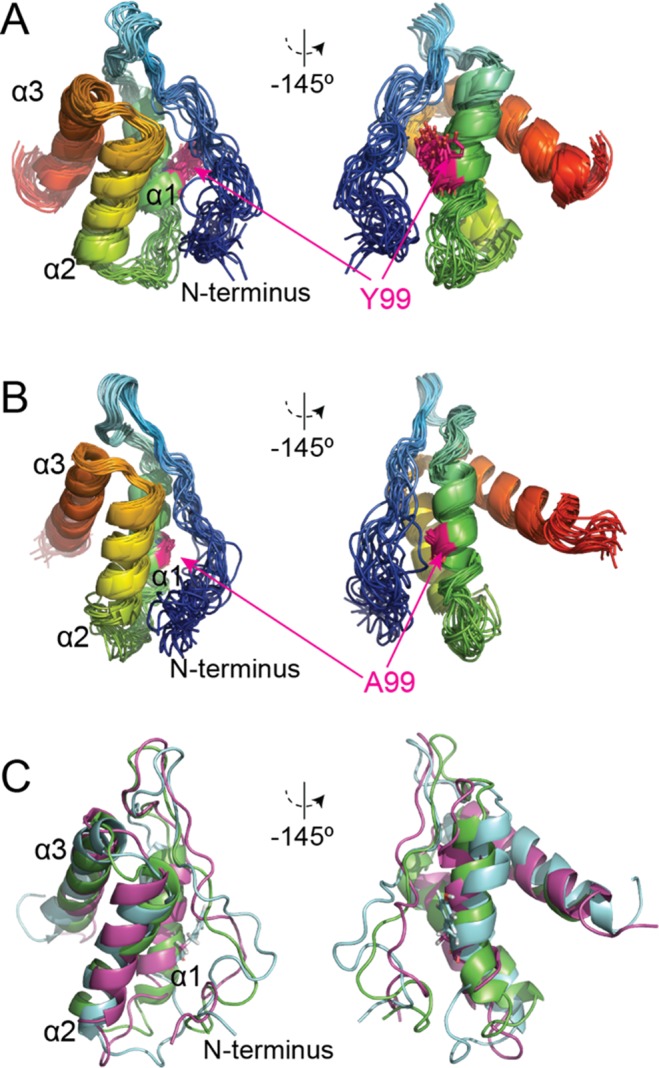
Details of MD1 and variant MD1_Y99A_ structures obtained by NMR spectroscopy. (**A**) Cartoon representations of the structure ensemble of the 20 best solution structures of MD1 and (**B**) MD1_Y99A_ variant. (**C**) Comparison of the representative NMR solution structures of MD1 (cyan) and MD1_Y99A_ (purple) with the crystal structure of MD1 from *B. glumae* (green) (PDB code 2ES4^10^).

So far, a high-resolution structure of Lif from *P. aeruginosa* does not exist; this is also true for each of the individual Lif domains. To obtain the first structural insights into this system and to investigate the effects of the critical Y99A mutation on the structure of the MD1 domain, we here solved the NMR solution-structure of the isolated MD1 domain (Fig. 4A, PDB code 5OVM; BMRB code 34175) as well as of the MD1_Y99A_ variant (Fig. 4B, PDB code 6GSF; BMRB code 34286).

Both MD1 and MD1_Y99A_ resemble a three α-helical bundle preceded by 27 N-terminal residues without clear secondary structure. Only minor structural differences were observed within each ensemble of 20 energetically most favorable structures for MD1 as well as for MD1_Y99A_, as indicated by RMSD_Cα_ of 1.3 ± 0.3 Å and 0.8 ± 0.2 Å, respectively. The obtained structures of the isolated MD1 variants are similar to the respective domain in the crystal structure of the Lif:LipA complex from *B. glumae* (Fig. 4C)^10^, showing that this domain adopts a stable fold, even when isolated and in the absence of a lipase.

Overall, both variants from *P. aeruginosa* exhibit rather similar 3D structures, with an RMSD_Cα_ of 2.4 Å, when comparing the MD1 and MD1_Y99A_ structural ensembles. This shows that the Y99A mutation does not alter the overall fold of MD1. Nevertheless, some differences are still visible when comparing both structures. These differences include (i) helix 2, which is slightly tilted in the MD1_Y99A_ variant as compared to MD1, as well as (ii) the degree of ‘disorder’ of the N-terminal coil including the loop interacting with helix 1. Yet, the second difference may be a direct consequence of limited distance restraints due to chemical exchange of the amide protons in this part of the protein.

**Figure 5:**
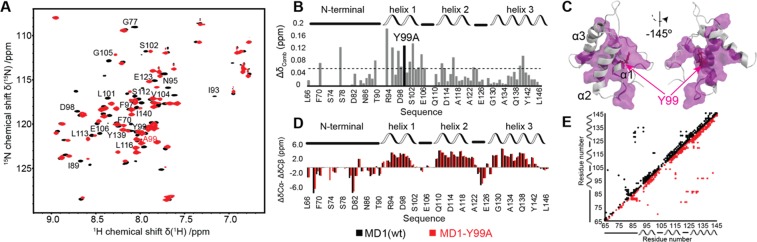
NMR-based structural comparison of MD1 and MD1_Y99A_. (**A**) ^1^H-^15^N-HSCQ spectra of MD1 (black) and MD1_Y99A_ (red). Labels correspond to the most affected residues due to the mutation. (**B**) Chemical shift perturbations induced by the mutation along the MD1 sequence and (**C**) mapped on the MD1 structure (purple, mutation site highlighted). (**D**) Comparison of ^13^C secondary chemical shifts of MD1 (black) and MD1_Y99A_ (red). Positive/negative values indicate α-helical/β-strand secondary structure. Random coil values should be zero. (**E**) Inter-residue distance restraints from NOEs for MD1 (black) and MD1_Y99A_ (red).

A comparison of the ^1^H-^15^N HSQC spectra for both MD1 variants reveals a rather high amount of chemical-shift perturbations induced by the point mutation (Fig. 5A,B). Mapping the strongest perturbations on the 3D structure suggests that the mutation does not only affect the chemical environment of its direct neighbors but does induce effects in several areas of the protein (Fig. 5C). This observation is consistent with the differences found in the structures for both variants, in particular the relative tilt of helix 2 in the protein core. Yet, when comparing the ^13^C chemical shifts (C_α_, C_β_), which are particularly sensitive to the secondary structure, only minor differences are found (Fig. 5D), again in accordance with the observed structural differences.

In agreement with the previously determined crystal structure of the homologous domain from *B. glumae*^10^, our data show that the N-terminal part does not exhibit a clear secondary structure motif. Furthermore, for larger parts of this region, the amide proton could not be detected, in line with elevated chemical exchange, indicating the absence of a hydrogen-bond network and/or the absence of protective steric effects provided by the remaining residues of the three-helix bundle.

However, our data also show that the N-terminal region is not completely disordered. On the one hand, this is confirmed by the rather low mobility seen in NMR dynamics data for several residues with observable amide protons (Fig. S7A–C). In fact, the loop connecting helix 1 and 2 appears to be more flexible than the N-terminal segment. On the other hand, secondary chemical shift analysis of ^13^C frequencies, which could be assigned for most N-terminal residues, differs substantially from a pure random coil character (Fig. 5D). Furthermore, clear inter-residue NOE correlations connect the N-terminal region to the three-helix bundle (Fig. 5E). Within the here detected parameters the features of the N-terminal region are similar in both MD1 variants.

To gain further insights into the effect of the Y99A mutation on the interaction of MD1 with LipA we acquired ^1^H,^15^N-HSQC spectra of MD1 and MD1_Y99A_ in the presence and absence of three-fold molar excess of LipA (Fig. S8). The data reveals a clearly noticeable signal decrease induced by the presence of LipA for MD1. In general, such a signal decrease can either indicate the formation of a tightly bound complex, which, however, is too large to be detected in the NMR spectrum. Therefore, only the reduced signal of the unbound state is detected (so-called NMR slow exchange regime). Based on fluorescence measurements obtained under comparable conditions it can be excluded that a tight complex is formed between MD1 and LipA (Fig. S5). Alternatively, the signal decrease can be explained by a transient interaction with exchange rates in the range of the NMR timescale, leading to peak broadening (so-called NMR intermediate exchange regime). Interestingly, the observed signal decrease is considerably stronger for MD1 as compared to MD1_Y99A_ suggesting that the mutation further reduces the interaction between MD1 and LipA. This observation is in line with the different behavior found in our inhibition assay (Fig. 2C) as well as the reduced LipA affinities observed for *s*Lif and *s*Lif_Y99A_ (Fig. 2D). Albeit the NMR data reveal minor but noticeable structural differences in MD1 structure due to the Y99A mutation and potential modulations in LipA affinity, it is at this point unclear how these differences can modulate Lif’s capability to fold LipA.

### *s*Lif_Y99A_ exerts a long-range effect on LipA which may destabilize the structure of the substrate-binding pocket

**Figure 6:**
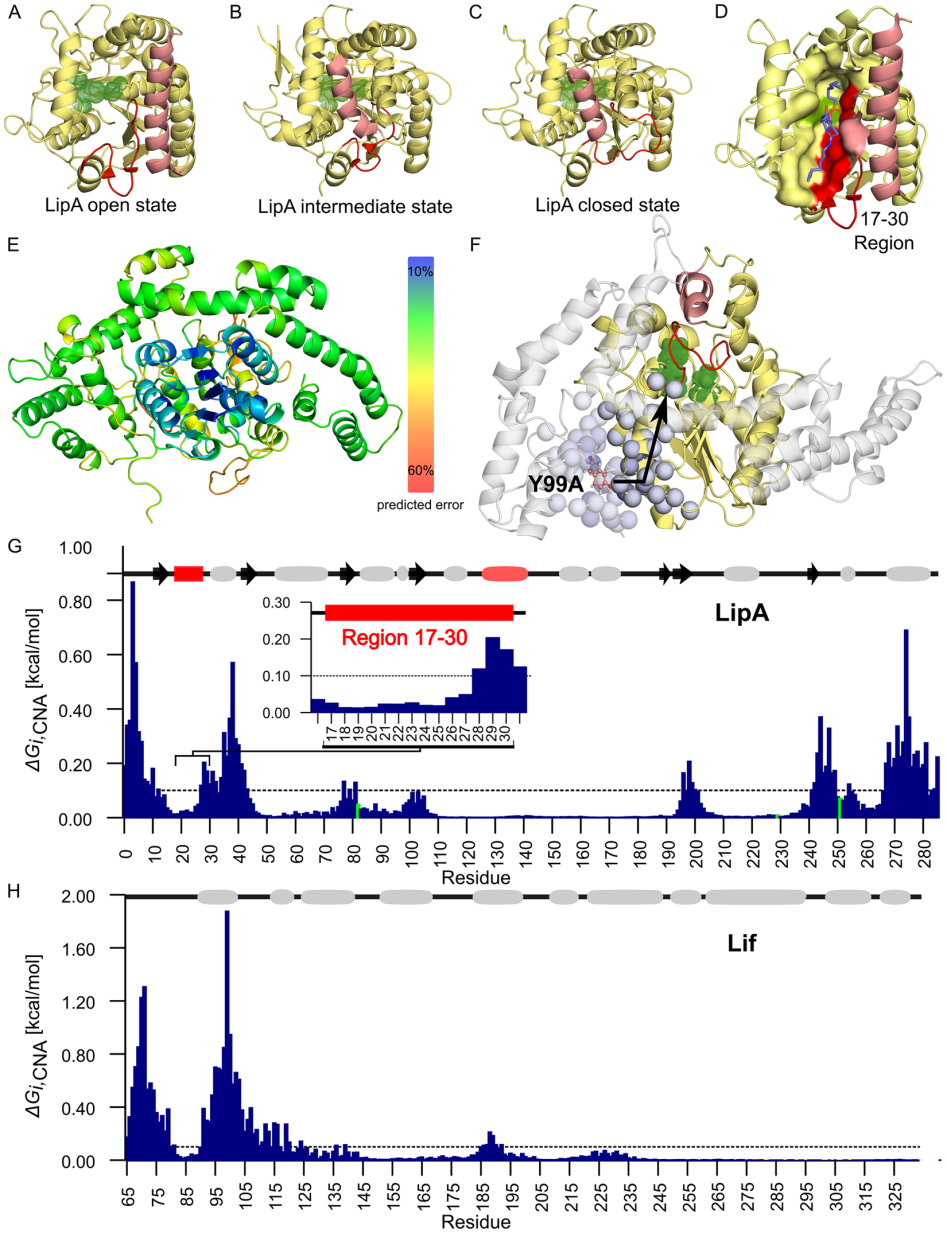
Influence of mutation Y99A in Lif on the structural stability of LipA. (**A**) Structure of *P. aeruginosa* LipA with OCP inhibitor bound in the active site crystallized in the open conformation (PDB code 1EX9^23^), in which the helix α5 (salmon) is moved away from the active site (catalytic triad residues S82, H251 and D229 shown in green). In this conformation, the active site is accessible for the substrate and LipA is enzymatically active. A short two-stranded β-sheet close to the active site is formed by residues 17–30 (red). (**B**) *B. glumae* lipase from the crystal structure of the Lif:LipA complex (PDB code 2ES4^10^). The lipase shows a two-stranded β-sheet (red), a characteristic feature of the open (active) conformation, nevertheless helix α5 (salmon) adopts a closed (inactive) conformation. This suggests a foldase-induced formation of a two-stranded β-sheet during activation of the lipase. (**C**) Crystal structure of *B. glumae* lipase crystallized in the closed conformation (PDB code 1QGE^50^) with helix α5 (salmon) covering the active site (residues as in panel A). In this conformation, a two-stranded β-sheet close to the active site is not formed. Residues 17–30 of *B. glumae* lipase, forming a two-stranded β-sheet, are indicated red. (**D**) Crystal structure of active *P. aeruginosa* LipA with inhibitor OCP bound in the active site (PDB code 1EX9). Region of residues 17–30 forms part of the active site (red), required for the binding of the ligand. (**E**) Homology model of the *P. aeruginosa* *s*Lif:LipA complex based on the structure of the *B. glumae* foldase-lipase complex (PDB code 2ES4^10^) used as a template. The coloring indicates the model quality assessment by TopScore^24,25^, with bluish colors representing less than 10% structural error. (**F**) CNA was applied on an ensemble of structures of the Lif:LipA complex generated from 10 independent MD simulations. Residues with $\Delta {G}_{i,{\rm{CNA}}}$ above a threshold of 0.1 kcal mol^−1^ are depicted as spheres on the Lif:LipA complex structure. Blue colors reflect predicted $\Delta {G}_{i,{\rm{CNA}}}$ values; the larger the value, the darker is the color. The black arrow indicates how the perturbation by Y99A mutation of Lif (pink, ball-and-stick representation) influences residues in LipA. Due to the decrease in the stability of the surrounding region of residues 17–30 in LipA, we speculate that the conformational changes required for the intermediate state of LipA on the way of activation is hampered upon Lif_Y99A_ mutation. The color code for helix α5, residues 17–30 and the active site is as in panel (A). (**G**) The histogram shows the per-residue $\Delta {G}_{i,{\rm{CNA}}}$ for LipA. The dashed line at 0.1 kcal mol^−1^ indicates the threshold above which residues are considered perturbed. The standard error of the mean is <0.05 kcal mol^−1^ for all residues. (**H**) Per-residue $\Delta {G}_{i,{\rm{CNA}}}$ shown for Lif, with the same threshold. The standard error of the mean is <0.05 kcal mol^−1^ for all residues.

To understand the possible role of the mutation Y99A for Lif-induced activation of LipA, we initially compared the X-ray structure of *P. aeruginosa* LipA in the open, active conformation (PDB code 1EX9) with the X-ray structures of *B. glumae* lipases in their closed, inactive conformations, one in the complex with its specific foldase (PDB code 2ES4) and the other in the unbound form (PDB code 1QGE) (Fig. 6A–C). In the open conformation, helix α5 is moved away from the active site, allowing substrates to access this site and a short two-stranded β-sheet close to the active site is formed by residues 21–22 and 25–26. The enclosing residues 17–30 shape the substrate-binding pocket^23^ (Fig. 6D). Because this β-sheet is not formed in the closed, unbound conformation of LipA, where helix α5 is occluding the active site, we postulated this structural element as a hallmark of the open and active LipA. Notably, the foldase-bound lipase, with an overall and active site structure mainly identical to those of unbound lipase^9,10^ does have a two-stranded β-sheet in the region of residues 17–30, yet helix α5 is in the closed conformation. The structural comparison thus indicates that the foldase induces the formation of the two-stranded β-sheet during activation of the lipase. Hence, the foldase-bound lipase can apparently be considered an intermediate conformation on its way to an open conformation, with residues 17–30 acting as a “loaded spring”.

The effect of mutation Y99A in Lif for lipase activation was further examined. We first generated a homology model of the *P. aeruginosa s*Lif:LipA complex, since no experimental structure of *P. aeruginosa* Lif has been reported so far. As a template structure, we used the structure of the *B. glumae* foldase-lipase complex (PDB code 2ES4) (see Materials and Methods section for details). The final model was assessed with our in-house model quality assessment program TopScore^24,25^ and found to be 68% correct for LipA and 52% correct for Lif (Fig. 6E). The final *s*Lif:LipA model was used as an input structure to perform ten independent all-atom MD simulations of 1.5 μs length each.

To investigate the influence of the Y99A mutation on the structural stability of LipA, we used an ensemble-based perturbation approach^26^ integrated into the constraint network analysis (CNA) approach, a method for analyzing rigidity and flexibility on constraint network representations of biomolecules, where the constraints are formed by covalent and noncovalent bonds^26^. In the perturbation approach, results from rigidity analyses are compared between a ground state and a perturbed network state. The change in biomolecular stability upon perturbation is quantified in terms of a residue-wise perturbation free energy $\Delta {G}_{i,{\rm{CNA}}}$ (Eq. 1). CNA was applied on the conformational ensemble of the *s*Lif:LipA complex generated from the above MD simulations, constituting the ground state (see Materials and Methods section for details). The perturbed state of the *s*Lif:LipA complex was generated by substituting Y99 with alanine, but keeping the structures of *s*Lif and LipA unchanged otherwise. We followed that approach because LipA strongly binds to both *s*Lif variants (Fig. 2B), suggesting that the respective complex structures are very similar in both cases. The computed changes in $\Delta {G}_{i,{\rm{CNA}}}$ (Eq. 1) were largest for residues 1–45, 197–202, 242–250 and 268–286 in LipA (Fig. 6F,G).

Notably, these affected residues form a narrow pathway that reaches the β-sheet-forming region including residues 28–30 (Fig. 6F,G), indicating that the mutation decreases the stability of substrate binding pocket (Fig. 6D). We speculate that this decrease in stability prevents the partial β-sheet formation of substrate binding pocket and, thus, disfavors LipA activation by *s*Lif_Y99A_. Finally, the Y99A mutation also affects the stability of a number of residues in sLif itself, especially around the mutation site, including residues 89–115, as well as residues 66–80 in MD1 (Fig. 6G). This observation is supported by NOE contacts identified in the NMR spectra (Fig. 5E) that connect these two regions, thus providing experimental validation of our computational strategy. In summary, our CNA results indicated that the Y99A mutation in MD1 of *s*Lif exerts a long-range influence on LipA structural stability that reaches the substrate-binding region that forms a β-sheet upon activation of LipA.

## Discussion

In this work, we studied the role of MD1 and of MD1’s residue Y99 on the activation of pre-active LipA by biochemical analysis, NMR and fluorescence spectroscopy, as well as molecular simulations. We show that the Y99A mutation in MD1 induces only minor changes in the domain’s overall structure. However, the activation of LipA induced by *s*Lif is inhibited by addition of MD1, which is not seen when using variant MD1_Y99A_ (Fig. 2C). Comparative CNA suggested that long-range network interactions span from Y99 of *s*Lif to the active site of LipA that are likely involved in LipA activation (Fig. 6).

MD1 was suggested as an essential domain for Lif activity as it contains an amino acid sequence motif conserved among all foldases^12^, which upon mutation leads to Lif inactivation^19^. We confirmed this finding by showing that the *P. aeruginosa s*Lif_Y99A_ variant carrying a single amino acid mutation in the foldase motif did not activate *P. aeruginosa* LipA, in contrast to *s*Lif (Fig. 2A).

Previously, Shibata and coworkers reported that *P. aeruginosa* Lif_Y99C_, Lif_Y99H_, Lif_S102R_ and Lif_R115C_ variants (all carrying mutations in the foldase motif) do not form complexes with LipA^19^. By contrast, we showed, using co-purification and fluorescence-based assays, that *s*Lif_Y99A_ binds pre-active LipA. Both proteins were co-purified by affinity chromatography and *s*Lif_Y99A_ in the presence of LipA yields an unfolding curve in nanoDSF very similar to that of *s*Lif in the presence of LipA but shifted compared to the unfolding curves of *s*Lif_Y99A_ and *s*Lif. Dissociation constants obtained from fluorescence binding assay reveal approximately 3-fold stronger binding of *s*Lif (*K*_D_ = 29 ± 9 nM) to pre-activated LipA than *s*Lif_Y99A_. The overall affinity is in the range previously reported for the complex from *B. glumae* (*K*_D_ = 5 nM)^10^.

The combined analysis of time-resolved fluorescence anisotropy and polarization-resolved FCS yields parameters, rotational correlation times and order parameters, that can be used to investigate shape and local flexibility of the fluorescently labelled proteins^20^.

Here, we introduce a stochastic labeling strategy of lysine residues by the anisotropy sensor BDP FL, to obtain insights into protein conformational flexibility and motions on the single-molecule level. We showed that, within the resolution of our approach, the Y99A mutation does not affect the shape and mobility of free *s*Lif (Fig. 3E, magenta and violet arrows). In light of these results, we expected that the mutation Y99A would not substantially alter the internal structure of *s*Lif. This assumption was strengthened by our observation that the melting temperatures of the two proteins measured by nanoDSF were similar (48.1 °C for *s*Lif and 50.5 °C for *s*Lif_Y99A_) (Fig. 3A). However, *s*Lif only contains a single fluorescent residue (W283) located in MD2, such that one cannot exclude that the Y99A mutation in MD1 may lead to structural changes that are not detected in the distant MD2.

With this in mind, we aimed at solving the 3D structures of *s*Lif and *s*Lif_Y99A_ by X-ray crystallography. Unfortunately, probably due to *s*Lif’s dynamic behavior^7^, all attempts have failed. We thus resorted to solving the structures of isolated *P. aeruginosa* MD1 and variant MD1_Y99A_ using solution NMR spectroscopy (Fig. 4). The MD1 solution structure provides the first experimental evidence that this domain adopts a stable tertiary structure even in the absence of LipA. The structure folds as a three α-helical bundle, stabilized by hydrophobic and aromatic residues (I93, F97, F100, L116, I120, L135, M136 and Y139) and is very similar to the structure of MD1 from the *B. glumae* Lif:LipA complex (Fig. 4). Furthermore, structural comparison of MD1 and MD1_Y99A_ revealed only minor structural changes, excluding that domain rearrangements or unfolding are the cause of the inactivation effect that is induced by the Y99A mutation. As such, the structural data alone does not allow to pinpoint the role of Y99 for LipA activation.

In the X-ray structure of *B. glumae* Lif:LipA complex^10^, residue Y91 (structurally equivalent to Y99 in *P. aeruginosa* Lif) contributes only little (~110 Å^2^ of solvent-accessible surface area) to the overall interface of ~5400 Å^2^ formed between the two proteins, which involves 65 Lif residues. Hence, it is not unexpected that *P. aeruginosa s*Lif_Y99A_ forms a complex with LipA, yet also this mutation decreases the affinity of *s*Lif towards LipA, which is confirmed by our fluorescence-based assay and NMR observation (Fig. S7). Indeed, we showed in *in vitro* experiments where isolated MD1 or MD1_Y99A_ compete with *s*Lif for LipA binding that MD1, but not MD1_Y99A_, inhibits LipA activation (Fig. 2C). pFCS and fluorescence anisotropy decay also highlight the important difference in mechanics of *s*Lif and *s*Lif_Y99A_ bound to LipA, where *s*Lif appears to be less mobile than in free form, while the local mobility of *s*Lif_Y99A_ is increased compared to *s*Lif (Fig. 3E, orange and blue arrows). Altogether, this indicates that for the mutant Y99A the MD1 domain does not, or only weakly, interact with LipA while the rest of sLif still interacts normally. Since DSF cannot sense effects in the MD1 domain (no Trp), this interpretation is with all data. Importantly, the increased flexibility of complexed *s*Lif_Y99A_ could also largely amplify the effect seen in the CNA.

Chemically denatured LipA, when refolded *in vitro*, adopts a globular pre-active conformation that shows a similar secondary structure content, intrinsic tryptophan fluorescence and susceptibility to proteolytic degradation as the native and active form of LipA^7,14^. This pre-active intermediate is converted to active LipA by addition of Lif. Apparently, structures of pre-active and native LipA are very similar, however, it is still unknown which structural changes in LipA are caused by Lif during activation. By analyzing the structures of native LipA from *P. aeruginosa* and *B. glumae*^9,10,23^ in different states, we observed conformational differences in a region close to the active site formed by residues 17–30 in lipases of *P. aeruginosa* and *B. glumae*. These residues are involved in the formation of a short, two-stranded β-sheet accompanying the opening of the lid-like α-helix covering the active site. We thus hypothesize that Lif may mediate LipA activation by promoting the formation of a short β-sheet formed by residues 17–30.

We tested this hypothesis by molecular simulations of *s*Lif and *s*Lif_Y99A_. CNA perturbation analyses of conformational ensembles generated by MD simulations starting from the comparative model of *P. aeruginosa* Lif:LipA complexes revealed that substitution Y99A destabilizes residues 28–30 involved in the formation of the short, two-stranded β-sheet in LipA (Fig. 6). We also identified a long-range network of interactions involving residues 1–45, 197–202, 242–250 and 268–286 in LipA that span from Y99 of Lif to the LipA β-sheet_17–30_ adjacent to the active site. We thus propose a mechanism of LipA activation based on the formation and stabilization of β-sheet_17–30_ in LipA through interactions with Lif. We recently showed by molecular dynamics simulations and potential of mean force computations that stabilization of β-sheet_17–30_ affects the dynamics of LipA’s lid and thereby LipA activation^27^. Such small structural changes upon LipA activation are expected given the previously observed similarity of pre-active and native LipA^7,14^.

In summary, our study reveals an intricate role of Y99 of the MD1 domain of *P. aeruginosa* Lif for LipA activation. Despite almost no influence on the global MD1 structure and weakly on *s*Lif global binding to LipA, the Y99A substitution hampers LipA activation, by the disruption of the mechanics of Lif:LipA complex. Molecular simulations suggest that, by long-range network interactions, Y99 supports the formation of a key secondary structure element in LipA on the way from pre-active to native LipA. Thus, our study for the first time provides insights at the atomistic level as to a potential mechanism of Lif-mediated pre-active-to-native LipA folding. This finding might spark further *in vitro* and *in vivo* studies to validate this putative mechanism.

## Experimental Procedures

### Cloning, protein production and purification

The expression plasmid encoding MD1 (pET-MD1) (Table S6) of Lif was created by PCR using Phusion DNA polymerase (Thermo Fischer Scientific) in whole plasmid amplification with mutagenic oligonucleotides (Table S6) designed for SLIC method^28^ and pEHTHis19^29^ plasmid as a template. For that purpose, amino acids 1–65 in Lif were deleted using primers Lif_dLinkVD_fw and Lif_dLinkVD_rv leaving the amino acid sequence MGHHHHHH before amino acid L66 of Lif. Subsequently, the sequence behind amino acid L146 was removed with the same method using the primers Lif_backbone_fw and Lif_MD1_rv. The expression plasmids pET-MD1_Y99A_ and pET-*s*Lif_Y99A_ respectively encoding MD1_Y99A_ and *s*Lif_Y99A_, the variants with mutation of Y99 to alanine, were created by whole plasmid PCR amplification with mutagenic oligonucleotide pair Lif_Y99A_fw/Lif_Y99A_rv designed for SLIC method and pET-MD1 or pEHTHis19 templates, respectively. *s*Lif, *s*Lif_Y99A_, MD1 and MD1_Y99A_ were expressed in *E. coli* BL21 (DE3) using respective expression plasmids as described previously^29^. For NMR studies, LB medium^30^ was replaced by M9 media supplemented with ^13^C-glucose and ^15^NH_4_Cl as sole carbon and nitrogen sources, respectively.

*s*Lif, *s*Lif_Y99A_, MD1 and MD1_Y99A_ variants carrying N-terminal His_6_-tag were purified by immobilized metal affinity chromatography using Ni-NTA resins (Qiagen) according to a protocol of Hausmann *et al*.^29^. Purified proteins were transferred to 20 mM sodium phosphate buffer (pH 7.4) by using PD10 column and concentrated to 500–1000 µM by using ultrafiltration device (Vivaspin) with a membrane of 5 kDa pore size. Protease inhibitor cocktail (0.1 x) (Sigma Aldrich) and 3 mM NaN_3_ were added to the final samples in order to ensure protein stability during long-term NMR experiments.

### Resonance assignment and structure calculation

NMR experiments were performed on Bruker Avance III HD^+^ spectrometers operating either at 600 or 700 MHz, both equipped with 5 mm inverse detection triple-resonance z-gradient cryogenic probes. Data was collected at 30 °C with sample concentrations between 450 and 900 µM in 20 mM sodium phosphate buffer pH 7.4 containing 10% (v/v) D_2_O, 0.01% sodium azide and 100 μM 4,4-dimethyl-4-silapentanesulfonic acid (DSS). All NMR spectra were processed with TOPSPIN 3.5 (Bruker BioSpin). DSS was used as a chemical shift standard and ^13^C and ^15^N data were referenced using frequency ratios as previously described^31^.

For the resonance assignment of MD1 and MD1_Y99A_, ^15^N- and ^13^C-edited HSQC (heteronuclear single-quantum coherence) and three-dimensional HNCO, HN(CA)CO, HN(CO)CACB and HNCACB experiments were performed to obtain the chemical shift assignments of the backbone atoms. Three-dimensional ^15^N- and ^13^C-NOESY-HSQC and (H)CCH-TOCSY, spectra were used for side-chain resonance assignment and NOE (nuclear Overhauser effect) measurements using acquisition parameters listed in Tables S2 and S3.

After assignment completion, CYANA2.1^32^ was used to analyze the peak data from the NOESY spectra in a semi-automated iterative manner. We used CARA 1.9.24a^33^ to automatically generate the NOE coordinates and intensities. The input data consisted of the amino acid sequence (of which we removed the histidine tag due to the lack of constraints), assigned chemical shift list, peak volume list and backbone dihedral angles (Φ and Ψ), which were derived from the TALOS+ server^34^ or with the CYANA script GridSearch^32^. The unambiguous NOEs assigned to a given pair of protons were converted into the upper limits by CYANA2.1^32^. No stereospecific assignments were introduced initially. In the final steps, 12 and 21 pairs of stereospecific restraints were introduced by CYANA2.1^32^ for MD1 and MD1_Y99A_, respectively.

The 20 conformers with the lowest final CYANA target function values were subjected to restrained energy-minimization as described in Pimenta *et al*.^35^ with the AMBER14 software package using the ff14SB force field^36^. The structures were placed in an octahedral periodic box of TIP3P water molecules^37^. The restrained energy minimization was then performed in three stages. First, the solute atoms were kept fixed with harmonic positional restraints with a force constant of 500 kcal mol^−1^ Å^−2^ to relax the solvent molecules. Subsequently, the entire system was relaxed after restraint removal. During the last stage, 1500 steps of NMR-restrained energy minimization were applied with a combination of steepest descent minimization followed by conjugate gradient minimization. A parabolic penalty function was used for the NOE upper distance restraints with a force constant of 20 kcal mol^−1^ Å^−2^. Finally, the geometric quality of the refined structures was analyzed with the Protein Structure Validation Software suite (version 1.5)^38^. Statistics for the NMR solution structures of MD1 and MD1_Y99A_ are given in Table 1.

**Table 1:** Statistics for the NMR structures of MD1 and MD1_Y99A_.

NMR distance and dihedral constraints	MD1	MD1_Y99A_
***Distance constraints***		
Total distance restraints from NOEs	964	1062
Short-range (|i − j| = 1)	676	694
Medium-range (|i − j|<5)	199	264
Long-range (|i − j|≥5)	89	104
*Total dihedral restraints*	104	219
**Structure statistics**		
***Violations (mean)***		
Distance constraints (Å)	0.0042 ± 0.0015	0.0055 ± 0.0015
Dihedral angle constraints (Å)	0.3001 ± 0.1139	0.2688 ± 0.0779
***CYANA target functions, Å***	0.70 ± 0.08	1.02 ± 0.15
***Average pairwise rmsd for residues 10***–***85***		
Backbone atoms	1.45 ± 0.22	1.37 ± 0.26
Heavy atoms	1.84 ± 0.16	1.88 ± 0.22
***Ramachandran’s plot analysis***		
Most favored regions	93.0%	90.4%
Additionally allowed regions	7.0%	6.7%
Generously allowed regions	0.0%	2.3%
Disallowed regions	0.0%	0.0%

### Accession numbers

The structural coordinates were deposited in the Protein Data Bank (PDB) under the accession codes 5OVM and 6GSF and the NMR data was deposited in the Biological Magnetic Resonance Data Bank (BMRB) under the accession numbers 34175 and 34286 for MD1 and MD1_Y99A_, respectively.

### MD1 and MD1_Y99A_ backbone dynamics

To gain insight into the backbone dynamics of MD1 and MD1_Y99A_ in the solution we measured the relaxation parameters *R*_1_, *R*_2_ and {^1^H}-^15^N-NOE (HetNOE) for both proteins at 35 °C. We used ^15^N-labelled samples at a concentration of 650 and 600 μM for MD1 and MD1_Y99A_, respectively. The solutions were prepared either in Tris-Glycine buffer pH 9 containing 10% (v/v) D_2_O, 0.01% sodium azide and 100 μM DSS or in 20 mM sodium phosphate buffer pH 7.4 containing 10% (v/v) D_2_O, 0.01% sodium azide and 100 μM DSS for MD1 and MD1_Y99A_, respectively. All data were collected in a Bruker Avance III HD^+^ 600 MHz spectrometer.

Backbone relaxation rates, *R*_1_ and *R*_2_, were determined by acquiring pseudo-3D spectra consisting of a series of 2D heteronuclear ^1^H-^15^N-HSQC experiments were the relaxation period varied. For the ^15^N longitudinal relaxation rates (*R*_1_), 12 time points were collected (0.02 s, 0.06 s, 0.1 s, 0.2 s, 0.4 s, 0.5 s, 0.6 s, 0.7 s, 0.8 s, 1.2 s, 1.5 s and 2 s). The spectra were acquired with 2048 points in ^1^H indirect dimension and 128 points in the ^15^N direct dimension and 8 scans. The spectral width was 7183.9 Hz in the ^1^H dimension and 1943.8 Hz in the ^15^N dimension and the relaxation delay was 1.5 s. The central frequency for proton was set on the solvent signal (2812.9 Hz) and for nitrogen was set on the center of the amide region (7535.96 Hz). For the ^15^N transverse relaxation rate (*R*_2_) 12 time points were collected (0.02 s, 0.03 s, 0.04 s, 0.06 s, 0.08 s, 0.1 s, 0.12 s, 0.14 s, 0.16 s, 0.2 s, 0.24 s and 0.28 s). The spectra were acquired in the same conditions as the above. The {^1^H}-^15^N-NOE steady-state NOE experiments were recorded with a relaxation delay of 10 s, with 8 transients in a matrix with 2048 data points in F2 and 256 increments in F1, in an interleaved manner, with alternating proton-pre-saturated and non-pre-saturated spectra. All data was processed with TopSpin 3.5 (Bruker BioSpin) and analyzed with CARA 1.9.24a^33^ and Relax 4.0.3^39^.

### MD1 and MD1_Y99A_ interaction with LipA

To investigate the effects of the mutation on the interaction with LipA, we followed the backbone signal intensity in ^1^H,^15^N-HSQC spectra of 120 μM ^15^N-labeled MD1 or MD1_Y99A_ in the presence and absence of 400 μM (unlabeled) LipA. All data was acquired at 10 °C, processed and analyzed with TopSpin 3.5 (Bruker BioSpin). Samples were prepared in Tris-Glycine buffer pH 9 containing 10% (v/v) D_2_O, 0.01% sodium azide and 100 μM DSS.

### *In vitro* activation of LipA with Lif

LipA, comprising residues S26-L311 without any affinity tag, was expressed in *E. coli* (BL21) DE3 using the plasmid pLipA-SS^29^. Cells expressing insoluble inclusion bodies of LipA were suspended in Tris-HCl buffer (100 mM, pH 7) containing 5 mM EDTA and 1 mM TCEP and disrupted by a French press. LipA inclusion bodies were collected by centrifugation at 10,000 *g* for 10 min and suspended in the same buffer. Centrifugation and wash steps were repeated three times to obtain purified LipA inclusion bodies. These were solubilized with Tris-HCl buffer containing 8 M urea at 37 °C for 1 h and remaining insoluble material was removed by centrifugation at 10,000 *g* for 10 min. Solubilized LipA was refolded by dilution with the TG buffer (5 mM glycine, 5 mM Tris, pH 9) containing an equimolar amount of *s*Lif and was incubated overnight at 4 °C.

### Lipase activity assay

The activity of LipA was spectrophotometrically monitored by the release of *p*-nitrophenolate from the standard lipase substrate *p*-nitrophenyl palmitate (*p*NPP, 1 mM) in 10 mM TG buffer containing 1 mM CaCl_2_^1^.

### Inhibition of LipA activation

LipA (50 nM) was incubated with MD1 or MD1_Y99A_ (0.2–1.0 µM) for 1 h at room temperature in a glass-coated 96-well microtiter plate (MTP) followed by addition of *s*Lif (50 nM). After agitating for 10 min at room temperature, *p*NPP lipase substrate (100 µL) was added to 100 µL of activated LipA in MTP and lipase activity was determined.

### Co-purification assays

*In vitro* Lif-LipA interaction studies were performed using *s*Lif and *s*Lif_Y99A_ variants carrying N-terminal His_6_-tags for immobilization onto Ni-NTA resins. First, the complexes of LipA (4 µM) with *s*Lif (4 µM) or *s*Lif_Y99A_ (4 µM) were formed in Tris-HCl buffer (10 mM Tris-HCl, pH 9) by incubation overnight at 4 °C followed by loading onto a Ni-NTA column and exhaustively washing with Tris-HCl buffer (10 mM, pH 9). The proteins bound to the column were eluted with Tris buffer (10 mM, pH 9) containing 500 mM imidazole. Elution fractions were analyzed by sodium dodecyl sulfate-polyacrylamide gel electrophoresis (SDS-PAGE) under denaturation conditions on 16% (w/v) gels^40^ followed by staining with Coomassie Brilliant Blue G250.

### Protein stability determination by differential scanning fluorimetry

LipA (2 µM) was incubated with *s*Lif (2 µM), *s*Lif_Y99A_ (2 µM), MD1 (2 µM) or MD1_Y99A_ (2 µM) overnight at 4 °C in TG buffer. The protein samples loaded into the measuring capillaries (Prometheus NT.Plex nanoDSF Grade Standard Capillary Chips) were heated from 15 °C to 95 °C (heating rate of 0.2 °C/min) and the intrinsic protein fluorescence was recorded at 330 nm and 350 nm using the Prometheus NT.Plex nanoDSF device (Nano Temper, Munich, Germany)^27^. The ratio of *F*_350nm_ and *F*_330nm_ and its first derivative were calculated by the PR.ThermControl software provided by the company.

### Model building of the P. aeruginosa Lif:LipA complex

The three-dimensional structure of *P. aeruginosa* Lif is currently unknown. Thus, a homology model of the *P. aeruginosa s*Lif:LipA complex was constructed using the structure of the *B. glumae* lipase:foldase complex (PDB code 2ES4) as a template (sequence identity/similarity: 39%/52%) for Lif and 41%/73% for LipA)^27^. The Phyre2 web server^41^ was used for homology modelling. The model obtained was energy minimized with the GROMOS96 43B1 force field as implemented in Swiss-PdbViewer^42^. After ten rounds of energy minimization, the C_α_ atoms of the models were superimposed on the template structure and the model with the lowest RMSD was taken for further studies^27^. The model obtained was evaluated by using our in-house model quality assessment program TopScore^24,25^. The correctness of the model is measured as the predicted global and local lDDT score^43^ compared to the native structure. The lDDT score compares all intra-molecular heavy-atom distances within two structures and, thus, is superposition-free. Two models are considered entirely different if all distances deviate by more than 4 Å and completely identical if all distances deviate by less than 0.5 Ȧ. Since the native structure is unknown, the score is predicted by a deep neural network which uses multiple sources of information as input. These include knowledge-based angle, distance and contact potentials, assessment of residue stereochemistry and atom clashes, model clustering and agreement between features predicted from the sequence and measured in the model, such as secondary structure, solvent accessibility and residue contacts. The deep neural network was trained on a large data-set of 660 protein targets totaling over 1.33 × 10^5^ models and over 1.9 × 10^7^ residues. The *P. aeruginosa* and *B. glumae* lipase:foldase complex structures show structural conservation of functionally important residues (Fig. S1), as for example the foldase motif residues (RXXFDY(F/C)L(S/T)A, X represents any amino acid) important for lipase activation^19^ and R343 related to the specificity of *B. glumae* foldase to bind its cognate lipase^10^. To validate our complex model, we mapped all conserved amino acids to the structures and found all of them at the interface of LipA and Lif, as expected.

### Molecular dynamics simulations

The refined model of *P. aeruginosa s*Lif:LipA complex was used as input structure for MD simulations. All-atom MD simulations were performed with the Amber 11 software package^44^ using the ff99SB force field^45^ as described in Ciglia *et al*.^46^. The *s*Lif:LipA complex was placed in an octahedral periodic box of TIP3P water molecules^37^ such that the smallest distance between the edges of the box and the closest solute atom is 11 Å. The SHAKE algorithm^46^ was applied to constrain bond lengths of hydrogen atoms and long-range electrostatic interactions were taken into account using the Particle Mesh Ewald method^47^. The time step was set to 2 fs with a non-bonded cut-off of 8 Å. The starting structures were first energy minimized by applying 50 steps of steepest descent minimization, followed by 450 steps of conjugate gradient minimization. During the minimization, the solute atoms were restrained applying decreasing harmonic potentials, with a force constant of 25 kcal mol^−1^ Å^−2^ initially, reduced to 5 kcal mol^−1^ Å^−2^ in the last round. For thermalization, the systems were heated from 100 K to 300 K in 50 ps of canonical (NVT)–MD simulations applying harmonic potentials with a force constant of 5 kcal mol^−1^ Å^−2^ on the solute atoms. Afterwards, MD simulations of 250 ps length were performed in the isothermal-isobaric ensemble (NPT) with the same harmonic potentials to adjust the density of the simulation box. Finally, the force constant of the harmonic restraints was reduced to zero during 100 ps of MD simulations in the NVT ensemble. For production, ten independent, unbiased MD simulations of 1.5 µs length were performed, totaling 15 µs of production runs. To ensure independence, production runs were carried out at temperatures of 300.0 K + *T*, where *T* was varied from 0.0 to 0.9 for each run, respectively.

### Constraint network analysis (CNA)

To detect changes in *s*Lif:LipA rigidity and flexibility upon Y99A mutation in *P. aeruginosa* Lif, we analyzed an ensemble of snapshots of *s*Lif-bound LipA in terms of a perturbation approach^26^ in a similar way as done by Milić *et al*.^25^. In short, first, an ensemble of 7,500 constraint network topologies was generated from MD snapshots of the proteins sampled at 2 ns intervals from the 10 MD simulations of the *s*Lif:LipA complex (see above). Second, altered bimolecular stability due to the *s*Lif_Y99A_ mutation is measured as per-residue perturbation free energy $\Delta {G}_{i,{\rm{CNA}}}$, following a linear response approximation (Eq. 1).

1 $$\Delta {G}_{i,{\rm{CNA}}}=\alpha (\langle {E}_{i,\,{\rm{CNA}}}^{perturbed}\rangle -\langle {E}_{i,\,{\rm{CNA}}}^{ground}\rangle )$$

Parameter α has been generally determined empirically and was set to 0.02 as in Pfleger *et al*.^26^ $\Delta {G}_{i,{\rm{CNA}}}$ was computed based on rigidity analyses performed with the CNA software package^26^ on ensembles of network topologies of the ground (*s*Lif) and perturbed (*s*Lif_Y99A_) states. Upon perturbation, about 19% of the residues in *s*Lif and 22% of the residues in LipA show altered stability characteristic according to $\Delta {G}_{i,{\rm{CNA}}}$ values > 0.1 kcal mol^−1^.

### Fluorescence labelling of Lif

Purified proteins *s*Lif, *s*Lif_Y99A_, MD1 and MD1_Y99A_ were transferred to 50 mM sodium phosphate buffer (pH 7.4) and concentration was adjusted to 70 μM. To label amino groups, Bodipy FL NHS ester (BDP FL; Lumiprobe), was dissolved in DMSO and added to the protein in 1:10 molar ratio to ensure labeling of single dye per protein molecule (obtained degree of labeling approximately 5%). Free dye was removed after overnight incubation at 4 °C by buffer exchange with Amicon Ultra-0.5 mL 10 K centrifugal filters (Merck-Milipore).

### Fluorescence measurements and data analysis

Steady-state fluorescence anisotropy *r*_steady-state_ and average translational diffusion time ‹*t*_*trans*_› were measured in the droplets on a cover-slide for 20 seconds to avoid changes of LipA concentrations due to protein adsorption. The concentrations of the labeled proteins were in the range of 1.2 ± 0.1 nM. The fluorescence signal was recorded on a custom-built confocal microscope^48^ with polarization-resolved detection with parallel- and perpendicular-polarized channels, *F*_p_(*t*) and *F*_s_(*t*). Anisotropy was calculated using equation:

2 $${r}_{steady-state}=\frac{{F}_{p}-{G}_{f}{F}_{s}}{{F}_{p}+2{G}_{f}{F}_{s}}$$

where *G*_f_ is the detection efficiency ratio between parallel and perpendicular channel.

The average translational diffusion time *t*_dif_ was calculated using Software Package for Multi-parameter Fluorescence Spectroscopy, Full Correlation and Multi-parameter Fluorescence Imaging^49^. Correlation curves *G*(*t*_c_) were approximated with 3-dimensional Gaussian diffusion model with 2 photophysical bunching terms:

3 $$G({t}_{c})=\frac{1}{N}{(1+\frac{{t}_{c}}{\langle {t}_{trans}\rangle })}^{-1}{(1+{(\frac{{\omega }_{0}}{{z}_{0}})}^{2}\times \frac{{t}_{c}}{\langle {t}_{trans}\rangle })}^{-\frac{1}{2}}+(1-{b}_{1}+{b}_{1}{e}^{-{t}_{c}/{t}_{b1}}-{b}_{2}+{b}_{2}{e}^{-{t}_{c}/{t}_{b2}})$$

here, the observation volume is approximated by a 3D-Gaussian volume with 1/*e*^2^ radii in the lateral (*ω*_0_) and axial direction (*z*_0_), with the particle number *N*, ‹*t*_*trans*_› is the apparent average translational diffusion time for the free and complexed *s*Lif and MD1 variants, respectively, *b*_1,2_ and *t*_*b1,b2*_ are amplitudes and times of the bunching terms.

The fraction of the complex x_complex_ was obtained from the linear combination of the fluorescence parameters of free *s*Lif-BDP FL and of *s*Lif-BDP FL in presence of >10 μM of LipA, assigned to be associated with Lif-LipA complex.

Polarization-resolved full fluorescence correlation spectroscopy was performed with a confocal laser scanning microscope (FV1000, Olympus, Germany) equipped with a single photon counting device with picosecond time-resolution (4 detectors, PD5CTC, Micro Photon Devices, Bolzano, Italy; counting electronics, HydraHarp400, PicoQuant, Berlin, Germany) at 23.5 ± 0.5 °C. The sample was excited by the continuous wave parked beam at 488 nm and the fluorescence *F* was collected in s- and p- polarized channels, *F*_s_(*t*) and *F*_p_(*t*), respectively. Full cross-correlation curves, *G*_p,p_(*t*_c_) and *G*_s,s_(*t*_c_), *G*_s,p_(*t*_c_) and *G*_p,s_(*t*_c_), were obtained according to Felekyan *et al*.^49^. Data were processed as previously described in Möckel *et al*.^20^.

Time-resolved fluorescence anisotropy decay curves were recorded using a FluoTime300 fluorescence lifetime spectrometer (PicoQuant, Berlin, Germany) equipped with a pulsed super continuum laser SuperK Extreme (NKT Photonics, Denmark) as a light source running at 15.61 MHz and a wavelength of 488 nm in a temperature-stabilized cell at 20.0 ± 0.1 °C. The fluorescence and anisotropy decays were recovered by global fitting of the sum (*F*_p_(*t*_c_) + 2*G*_f_*F*_s_(*t*_c_)) and difference (*F*_p_(*t*_c_) − *G*_f_*F*_s_(*t*_c_)) curves as previously described (Eq. S2a,b)^20^.

### *In vitro* binding of LipA and fluorescently labelled sLif

Labelled *s*Lif/ *s*Lif_Y99A_ –BDP FL (1.2 ± 0.1 nM of BFL, total concentration ${c}_{sLif}^{0}$ approximately 24 nM)) was incubated overnight at 4 °C with various concentrations of LipA (0–50 μM) in 10 mM glycine buffer (10 mM, pH 9) in protein low binding tubes (Sarstedt AG). Equilibrium dissociation constant *K*_D_ was fitted using 1:1 binding model:

4 $${x}_{complex}=\frac{1}{{c}_{sLif}^{0}}(\frac{{K}_{D}+{c}_{sLif}^{0}+{c}_{LipA}^{0}}{2}-\sqrt{{(\frac{{K}_{D}+{c}_{sLif}^{0}+{c}_{LipA}^{0}}{2})}^{2}-{c}_{sLif}^{0}\cdot {c}_{LipA}^{0}})$$

where x_complex_ is a fraction of Lif in complex with LipA, KD is a dissociation constant and ${c}_{sLif}^{0}$ and ${c}_{LipA}^{0}$ are the total concentrations of *s*Lif and LipA.

## Supplementary information

Supplementary information.

## References

[1] Jaeger KE, Kovacic F (2014). Determination of lipolytic enzyme activities. Methods Mol. Biol..

[2] Bleves S (2010). Protein secretion systems in *Pseudomonas aeruginosa*: A wealth of pathogenic weapons. Int. J. Med. Microbiol..

[3] Jaeger KE, Kharazmi A, Hoiby N (1991). Extracellular lipase of *Pseudomonas aeruginosa*: biochemical characterization and effect on human neutrophil and monocyte function *in vitro*. Microb. Pathog..

[4] Konig B, Jaeger KE, Konig W (1994). Induction of inflammatory mediator release (12-hydroxyeicosatetraenoic acid) from human platelets by *Pseudomonas aeruginosa*. Int. Arch. Allergy Immunol..

[5] Konig B, Jaeger KE, Sage AE, Vasil ML, Konig W (1996). Role of *Pseudomonas aeruginosa* lipase in inflammatory mediator release from human inflammatory effector cells (platelets, granulocytes, and monocytes. Infect. Immun..

[6] Jaeger KE (1994). Bacterial lipases. FEMS Microbiol. Rev..

[7] Pauwels K, Sanchez del Pino MM, Feller G, Van Gelder P (2012). Decoding the folding of *Burkholderia glumae* lipase: folding intermediates en route to kinetic stability. PLoS One.

[8] El Khattabi M, Ockhuijsen C, Bitter W, Jaeger KE, Tommassen J (1999). Specificity of the lipase-specific foldases of Gram-negative bacteria and the role of the membrane anchor. Mol. Gen. Genet..

[9] Noble ME, Cleasby A, Johnson LN, Egmond MR, Frenken LG (1993). The crystal structure of triacylglycerol lipase from *Pseudomonas glumae* reveals a partially redundant catalytic aspartate. FEBS Lett..

[10] Pauwels K (2006). Structure of a membrane-based steric chaperone in complex with its lipase substrate. Nat. Struct. Mol. Biol..

[11] Shibata H, Kato H, Oda J (1998). Molecular properties and activity of amino-terminal truncated forms of lipase activator protein. Biosci. Biotechnol. Biochem..

[12] Rosenau F, Tommassen J, Jaeger KE (2004). Lipase-specific foldases. Chembiochem.

[13] Nagradova N (2007). Enzymes catalyzing protein folding and their cellular functions. Curr. Protein Pept. Sci..

[14] El Khattabi M, Van Gelder P, Bitter W, Tommassen J (2000). Role of the lipase-specific foldase of *Burkholderia glumae* as a steric chaperone. J. Biol. Chem..

[15] Ihara F, Okamoto I, Akao K, Nihira T, Yamada Y (1995). Lipase modulator protein (LimL) of *Pseudomonas* sp. strain 109. J. Bacteriol..

[16] Hobson AH, Buckley CM, Jorgensen ST, Diderichsen B, McConnell DJ (1995). Interaction of the *Pseudomonas cepacia* DSM3959 lipase with its chaperone, LimA. J. Biochem..

[17] Eder J, Rheinnecker M, Fersht AR (1993). Folding of subtilisin BPN’: characterization of a folding intermediate. Biochem..

[18] Dodson KW, Jacob-Dubuisson F, Striker RT, Hultgren SJ (1993). Outer-membrane PapC molecular usher discriminately recognizes periplasmic chaperone-pilus subunit complexes. Proc. Natl Acad. Sci. USA.

[19] Shibata H, Kato H, Oda J (1998). Random mutagenesis on the *Pseudomonas* lipase activator protein, LipB: exploring amino acid residues required for its function. Protein Eng..

[20] Mockel C (2019). Integrated NMR, fluorescence, and molecular dynamics benchmark study of protein mechanics and hydrodynamics. J. Phys. Chem. B.

[21] Tsytlonok M (2019). Dynamic anticipation by Cdk2/Cyclin A-bound p27 mediates signal integration in cell cycle regulation. Nat. Commun..

[22] Ortega A, Amoros D, Garcia de la Torre J (2011). Prediction of hydrodynamic and other solution properties of rigid proteins from atomic- and residue-level models. Biophys. J..

[23] Nardini M, Lang DA, Liebeton K, Jaeger KE, Dijkstra BW (2000). Crystal structure of *Pseudomonas aeruginosa* lipase in the open conformation. The prototype for family I.1 of bacterial lipases. J. Biol. Chem..

[24] Mulnaes D, Gohlke H (2018). TopScore: Using deep neural networks and large diverse data sets for accurate protein model quality assessment. J. Chem. Theory Comput..

[25] Milic D (2018). Recognition motif and mechanism of ripening inhibitory peptides in plant hormone receptor ETR1. Sci. Rep..

[26] Pfleger C (2017). Ensemble- and rigidity theory-based perturbation approach to analyze dynamic allostery. J. Chem. Theory Comput..

[27] Verma, N., Dollinger, P., Kovacic, F., Jaeger, K.-E., Gohlke, H. The membrane-integrated steric chaperone Lif facilitates active site opening of *Pseudomonas aeruginosa* lipase A. *J. Comp. Chem.* **41**, 500–512 (2020). 10.1002/jcc.26085 31618459

[28] Li MZ, Elledge SJ (2007). Harnessing homologous recombination *in vitro* to generate recombinant DNA via SLIC. Nat. Methods.

[29] Hausmann S, Wilhelm S, Jaeger KE, Rosenau F (2008). Mutations towards enantioselectivity adversely affect secretion of *Pseudomonas aeruginosa* lipase. FEMS Microbiol. Lett..

[30] Bertani G (1951). Studies on lysogenesis. I. The mode of phage liberation by lysogenic. Escherichia coli, J. Bacteriol..

[31] Wishart DS, Bigam CG, Holm A, Hodges RS, Sykes BD (1995). 1H, 13C and 15N random coil NMR chemical shifts of the common amino acids. I. Investigations of nearest-neighbor effects. J. Biomol. NMR.

[32] Guntert P (2004). Automated NMR structure calculation with CYANA. Methods Mol. Biol..

[33] Rochus, L. J. K. *The Computer Aided Resonance Assignment Tutorial*, CANTINA Verlag, Goldau, Switzerland. (2004)

[34] Cornilescu G, Delaglio F, Bax A (1999). Protein backbone angle restraints from searching a database for chemical shift and sequence homology. J. Biomol. NMR.

[35] Pimenta J (2013). NMR solution structure and SRP54M predicted interaction of the N-terminal sequence (1-30) of the ovine Doppel protein. Peptides.

[36] Maier JA (2015). ff14SB: Improving the accuracy of protein side chain and backbone parameters from ff99SB. J. Chem. Theory Comput..

[37] Jorgensen WL, Chandrasekhar J, Madura JD (1983). Comparison of simple potential functions for simulating liquid water. J. Chem. Phys..

[38] Bhattacharya A, Tejero R, Montelione GT (2007). Evaluating protein structures determined by structural genomics consortia. Proteins.

[39] Bieri M, d’Auvergne EJ, Gooley PR (2011). relaxGUI: a new software for fast and simple NMR relaxation data analysis and calculation of ps-ns and mus motion of proteins. J. Biomol. NMR.

[40] Laemmli UK (1970). Cleavage of structural proteins during the assembly of the head of bacteriophage T4. Nat..

[41] Kelley LA, Sternberg MJ (2009). Protein structure prediction on the Web: a case study using the Phyre server. Nat. Protoc..

[42] Guex N, Peitsch MC (1997). SWISS-MODEL and the Swiss-PdbViewer: an environment for comparative protein modeling. Electrophoresis.

[43] Mariani V, Biasini M, Barbato A, Schwede T (2013). lDDT: a local superposition-free score for comparing protein structures and models using distance difference tests. Bioinforma..

[44] Case DA (2005). The Amber biomolecular simulation programs. J. Comput. Chem..

[45] Hornak V (2006). Comparison of multiple Amber force fields and development of improved protein backbone parameters. Proteins.

[46] Ciglia Emanuele, Vergin Janina, Reimann Sven, Smits Sander H. J., Schmitt Lutz, Groth Georg, Gohlke Holger (2014). Resolving Hot Spots in the C-Terminal Dimerization Domain that Determine the Stability of the Molecular Chaperone Hsp90. PLoS ONE.

[47] Darden T, York D, Pedersen L (1993). Particle mesh Ewald - an N.Log(N) method for Ewald sums in large systems. J. Chem. Phys..

[48] Sisamakis E, Valeri A, Kalinin S, Rothwell PJ, Seidel CAM (2010). Accurate single-molecule FRET studies using multiparameter fluorescence detection. Method. Enzymol..

[49] Felekyan S., Kühnemuth R., Kudryavtsev V., Sandhagen C., Becker W., Seidel C. A. M. (2005). Full correlation from picoseconds to seconds by time-resolved and time-correlated single photon detection. Review of Scientific Instruments.

[50] Lang D (1996). Crystal structure of a bacterial lipase from *Chromobacterium viscosum* ATCC 6918 refined at 1.6 angstroms resolution. J. Mol. Biol..

