# Supplementary material for: Structural and dynamic insights revealing how lipase binding domain MD1 of Pseudomonas aeruginosa foldase affects lipase activation
Source: Sci Rep. 2020 Feb 27;10:3578. doi: 10.1038/s41598-020-60093-4 (PMC7046727; doi:10.1038/s41598-020-60093-4)
Supplement: Supplementary file 1 — Supplementary information. [file 41598_2020_60093_MOESM1_ESM.pdf]

SUPPORTING INFORMATION

Structural and dynamic insights revealing how lipase binding domain MD1 of *Pseudomonas aeruginosa* foldase affects lipase activation

Aldino Viegas[1,$,#], Peter Dollinger[2,$], Neha Verma[3,$], Jakub Kubiak[5], Thibault Viennet[1,4], Claus A.M. Seidel[5], Holger Gohlke[3,4,6,*], Manuel Etzkorn[1,4,7*], Filip Kovacic[2], and Karl-Erich Jaeger[2,8,*]

Running title: **Structural insights into foldase-mediated lipase activation**

From the [1]Institute of Physical Biology, Heinrich Heine University Düsseldorf, 40225 Düsseldorf, Germany; [2]Institute of Molecular Enzyme Technology, Heinrich Heine University Düsseldorf, 52425 Jülich, Germany; [3]Institute of Pharmaceutical and Medicinal Chemistry, Heinrich Heine University Düsseldorf, 40225 Düsseldorf, Germany; [4]Institute for Complex Systems ICS-6: Structural Biochemistry, Forschungszentrum Jülich GmbH, 52425 Jülich, Germany; [5]Institute of Molecular Physical Chemistry, Heinrich Heine University Düsseldorf, 40225 Düsseldorf, Germany; [6]John von Neumann Institute for Computing (NIC) and Jülich Supercomputing Centre (JSC), Forschungszentrum Jülich GmbH, 52425 Jülich, Germany; [7]JuStruct: Jülich Center for Structural Biology, Forschungszentrum Jülich GmbH, 52425 Jülich, Germany; [8]Institute of Bio- and Geosciences IBG-1: Biotechnology, Forschungszentrum Jülich GmbH, 52425 Jülich, Germany

[#]Present address: Departamento de Química, Faculdade de Ciências e Tecnologia UCIBIO-REQUIMTE, Universidade Nova de Lisboa, 2829-516 Caparica, Portugal

*To whom correspondence should be addressed: Karl-Erich Jaeger: Institute of Molecular Enzyme Technology, Heinrich Heine University Düsseldorf, D-52425, Jülich, Wilhelm Johnen Str. 1, karl-erich.jaeger@fz-juelich.de; Tel. (+49)02461616966; Fax. (+49)02461612490. Manuel Etzkorn: Institute of Pharmaceutical and Medicinal Chemistry, Heinrich Heine University Düsseldorf, D-40225, Düsseldorf, Universitätsstr. 1; manuel.etzkorn@hhu.de; Tel. (+49)2118112023; Fax. (+49)2118113847. Holger Gohlke: Institute of Pharmaceutical and Medicinal Chemistry, Heinrich Heine University Düsseldorf, D-40225, Düsseldorf, Universitätsstr. 1; gohlke@uni-duesseldorf.de; Tel. (+49) 2118113662; Fax. (+49)2118113847.

[$]These authors contributed equally to this work.

Supplementary Content

Figure S1	Sequence alignment of foldases from *B. glumae* and *P. aeruginosa*.
Figure S2	SDS-PAGE analysis of purified *P. aeruginosa* MD1 and $MD1_{Y99A}$ used for solution NMR analysis.
Figure S3	Melting curves of MD1, $MD1_{Y99A}$, pre-active LipA alone and after incubation with MD1 and $MD1_{Y99A}$
Figure S4	Steady-state fluorescence anisotropy titration of *s*Lif, *s*Lif_{Y99A}, MD1 and $MD1_{Y99A}$ with LipA
Figure S5	Interaction of fluorescently labeled MD1 and $MD1_{Y99A}$ with LipA
Figure S6	Time-resolved fluorescence anisotropy decay fits of *s*Lif and *s*Lif_{Y99A}
Figure S7	^{15}N backbone relaxation data R_1, R_2 and heteronuclear {^{1}H}-^{15}N NOE (HetNOE)
Figure S8	^{1}H-^{15}N HSQC spectra of isotope labelled variants of MD1 in the absence (black) and presence of 3-fold molar excess of unlabeled LipA.
Table S1	Steady-state fluorescence anisotropy of *s*Lif labeled with BDP FL
Table S2	Average translational diffusion time $\langle t_{trans} \rangle$ of *s*Lif_{Y99A} labeled with BDP FL
Table S3	Fluorescence anisotropy decay fit parameters for MD1 and $MD1_{Y99A}$ labeled with BDP FL
Table S4	Fluorescence anisotropy decay fit parameters *s*Lif and *s*Lif_{Y99A} labeled with BDP FL
Table S5	pFCS fit parameters for *s*Lif and *s*Lif_{Y99A}
Table S6	List of used bacterial strains, plasmids and oligonucleotides.
Table S7	Acquisition parameters of the spectra used for MD1 resonance assignment and structure calculation.
Table S8	Acquisition parameters of the spectra used for $MD1_{Y99A}$ resonance assignment and structure calculation.
Section S1	pFCS analysis
Section S2	Fluorescence anisotropy decay analysis
Section S3	Determination of half-inactivation temperature

Supplementary Figures

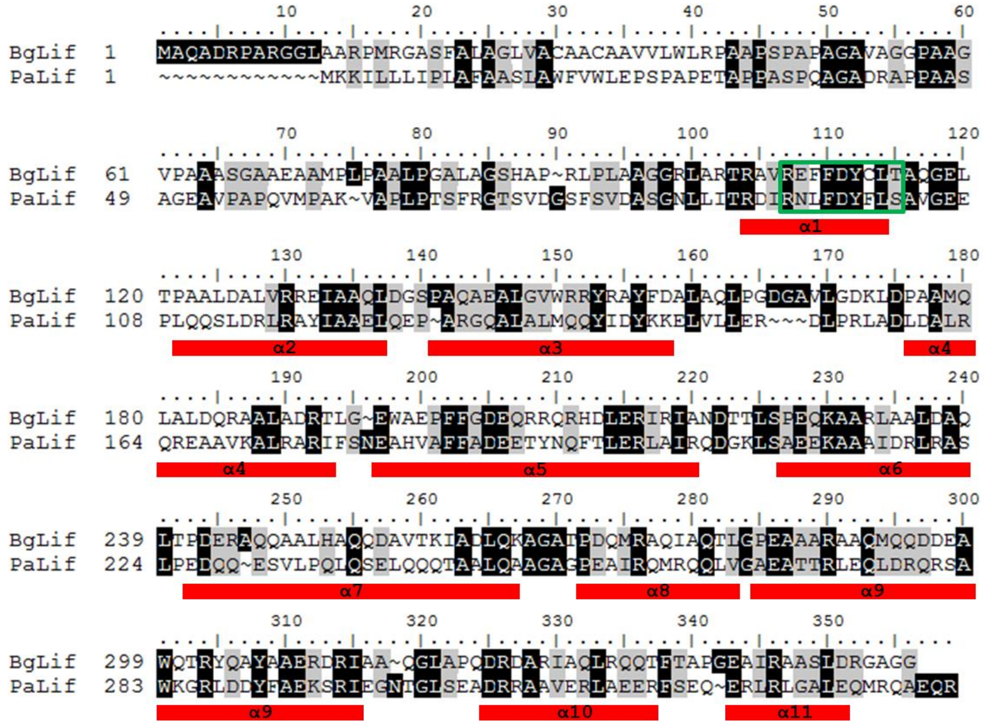

Figure S1 Sequence alignment of foldases from *B. glumae* and *P. aeruginosa*. BgLif and PaLif share 39% identical and 52% similar amino acids, shown on a black and gray background, respectively. Sequence similarity of *P. aeruginosa* and *B. glumae* TMD, VLD, MD1, EHD and MD2 is 31%, 44%, 52%, 46% and 48%, respectively. The red bars underneath the alignment indicate α-helices of the experimentally determined BgLif structure. The conserved RxxFDY(F/C)L(S/T)A foldase motif is indicated with a green frame. Numbers in front of the sequences indicate amino acid positions in the sequences.

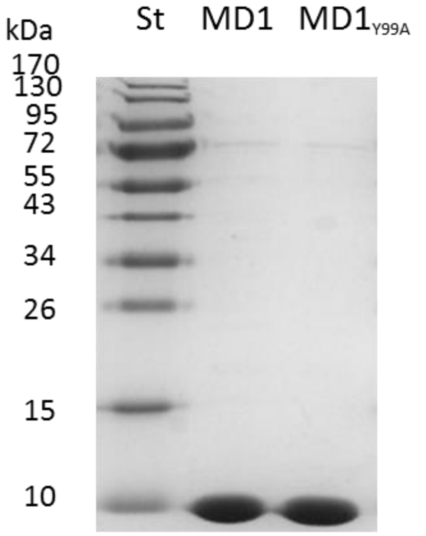

Figure S2 SDS-PAGE analysis of purified *P. aeruginosa* MD1 and MD1$_{Y99A}$ used for solution NMR analysis. The gel (16 %) was stained with Coomassie Brilliant Blue G250. Molecular weights of standard proteins (St) are indicated on the left-hand side.

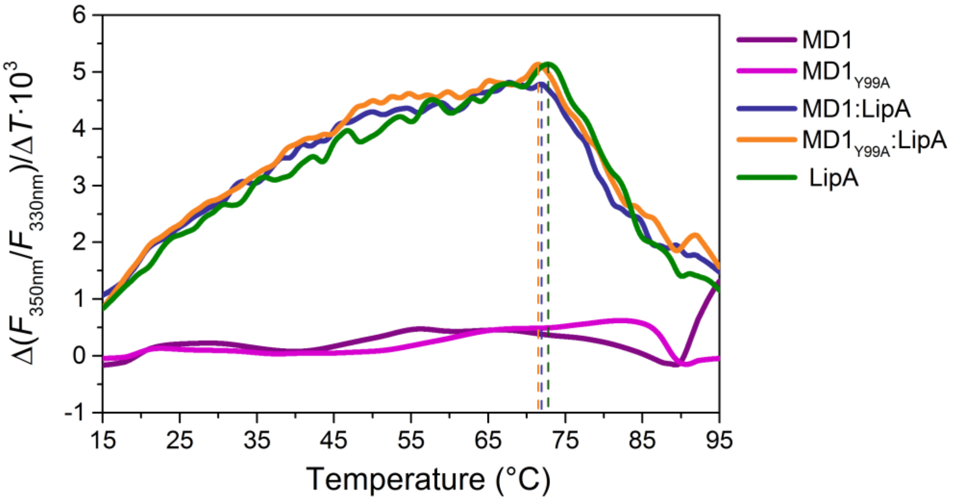

Figure S3 Melting curves of MD1, $MD1_{Y99A}$, pre-active LipA alone and after incubation with MD1 and $MD1_{Y99A}$ obtained by fluorescence measurement with nanoDSF. Vertical dashed lines indicate melting temperatures of LipA not affected significantly by incubation with MD1 or $MD1_{Y99A}$.

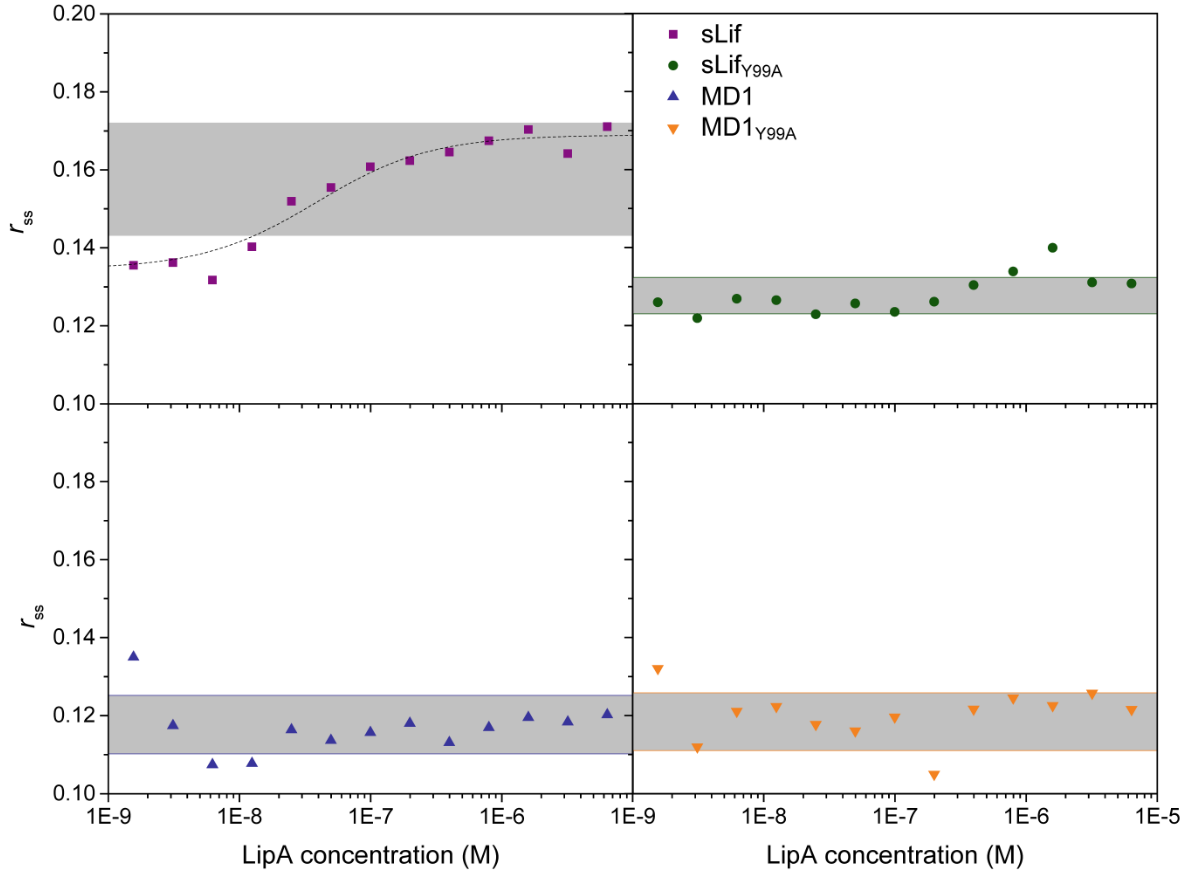

Figure S4 Steady-state fluorescence anisotropy $r_{steady\text{-}state}$ titration of sLif, sLif$_{Y99A}$, MD1 and MD1$_{Y99A}$ labelled with BDP FL NHS ester with different concentrations of LipA. Gray bar indicates average steady-state anisotropy ± the standard deviation. Steady-state anisotropy is a good marker of *s*Lif binding to LipA. However in case of sLif$_{Y99A}$, MD1 and MD1$_{Y99A}$ there is no observable change in this parameter at LipA concentrations of up to 10 µM. Steady-state anisotropy is a product of fluorescence decay and fluorescence anisotropy decay $\boldsymbol{r_{steady-state}} = \int_0^\infty \boldsymbol{F(t_c)r(t_c)dt_c} \,/ \int_0^\infty \boldsymbol{F(t_c)dt_c}$, which is sensitive i.a. to the dye flexibility and size of the rotating molecule. Stable steady-state anisotropy marks the lack of formation of complex between labelled molecules and LipA or change in other parameters influencing $r_{steady\text{-}state}$.

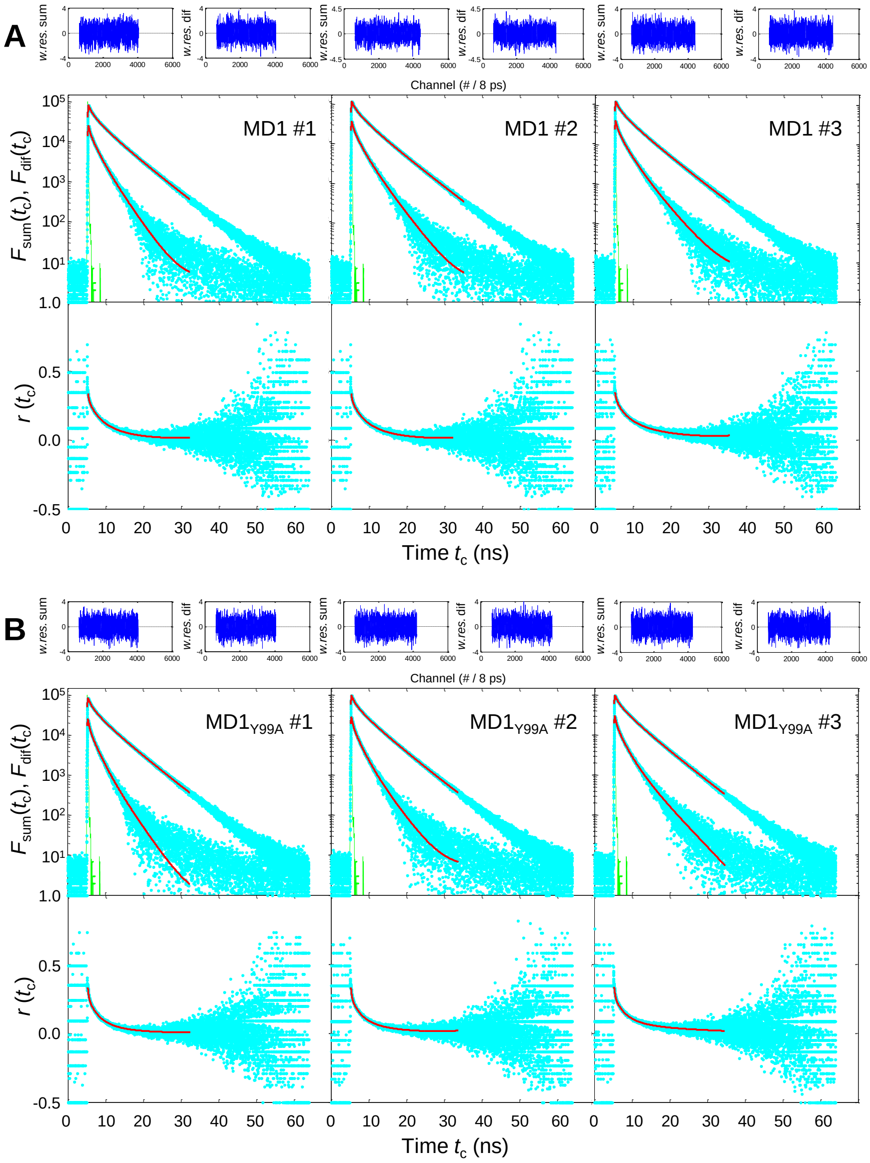

Figure S5. Interaction of fluorescently labeled MD1 (A) and MD1$_{Y99A}$ (B) with LipA: condition #1 - 0.1 μM MD1/MD1$_{Y99A}$; condition #2 - 0.1 mM MD1/MD1$_{Y99A}$ (NMR conditions); and condition #3 - 0.1 mM MD1/MD1$_{Y99A}$ and 0.4 mM LipA (NMR conditions). Joint time-resolved fluorescence anisotropy decay fits (sum and difference: ($F_{sum}(t_c)$ = $F(t_c)$, $F_{dif}(t_c)$ = $\mathrm{F}(t_c) \cdot r(t_c)$). Fit parameters are collected in supplementary **Table S3**. MD1 and MD1$_{Y99A}$ show similar hydrodynamic properties and both experience slower rotation at 0.1 mM concentrations (condition #2), which canbe explained by increased viscosity (factor 1.2 compared to condition #1). Assuming global rotational correlation time of MD1:LipA complex of ρ_{global} = 32 ns, a fit of fluorescence anisotropy decay requires approx. 20% of such complex fraction in case of both MD1 and MD1$_{Y99A}$ in presence of 0.4 mM LipA (condition #3).

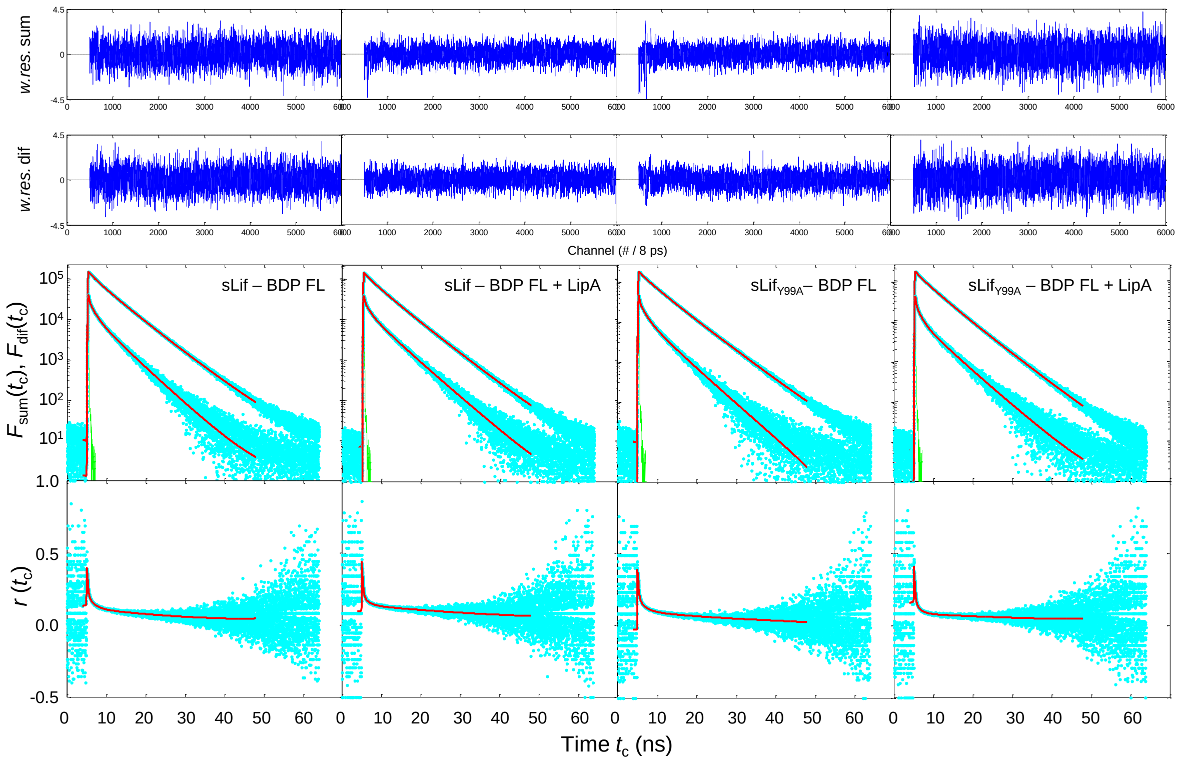

Figure S6 Time-resolved fluorescence anisotropy decay fits (sum and difference fit: $F_{\text{sum}}(t_c) = F(t_c)$, $F_{\text{dif}}(t_c) = F(t_c) \cdot r(t_c)$) of *s*Lif and *s*Lif$_{\text{Y99A}}$ labelled with BDP FL NHS ester in absence and presence of 40 µM sLipA. Amplitude of global rotational correlation time (r_3) is similar for *s*Lif and *s*Lif:LipA complex, while global rotational correlation time ρ_3 changest from approx. 33 ns to 50 ns. In case of *s*Lif$_{\text{Y99A}}$ ρ_3 shows similar behaviour but amplitude of global rotation (r_3) decreases significantly from 0.10 to 0.06. Fit parameters are collected in **Table S4**.

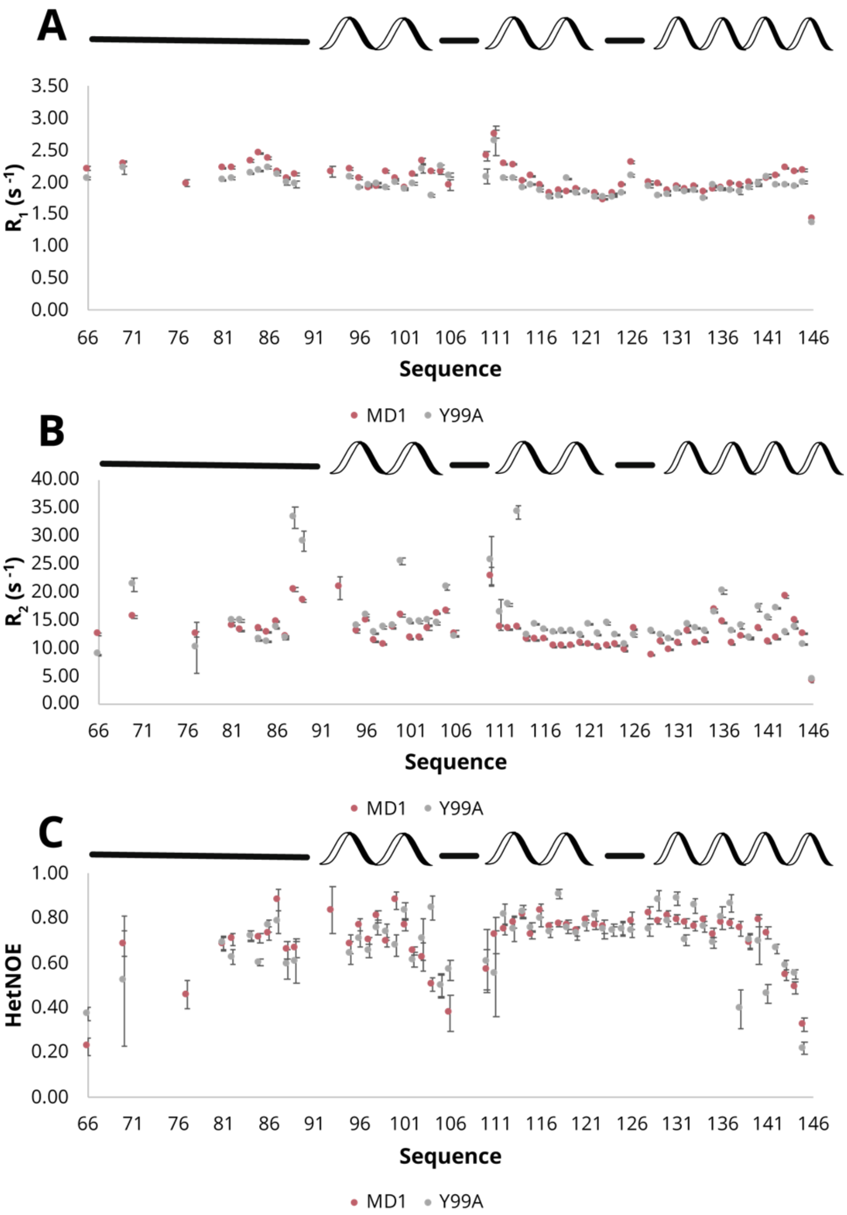

Figure S7 ^{15}N backbone relaxation data R_1, R_2 and heteronuclear {^{1}H}-^{15}N NOE (HetNOE) measured for MD1 (red) and MD1$_{Y99A}$ (grey) are shown in A, B, C, respectively. Secondary structure elements are illustrated on top of the figure. Data was acquired using ^{15}N-labelled samples at a concentration of 650 and 600 µM for MD1 and MD1$_{Y99A}$, respectively. The solutions were prepared either in Tris-Glycine buffer pH 9 containing 10% (v/v) D_2O, 0.01% sodium azide and 100 µM DSS or in 20 mM sodium phosphate buffer pH 7.4 containing 10% (v/v) D_2O, 0.01% sodium azide and 100 µM DSS for MD1 and MD1$_{Y99A}$, respectively. All data were collected in a Bruker Avance III HD$^+$ 600 MHz spectrometer, at 35°C.

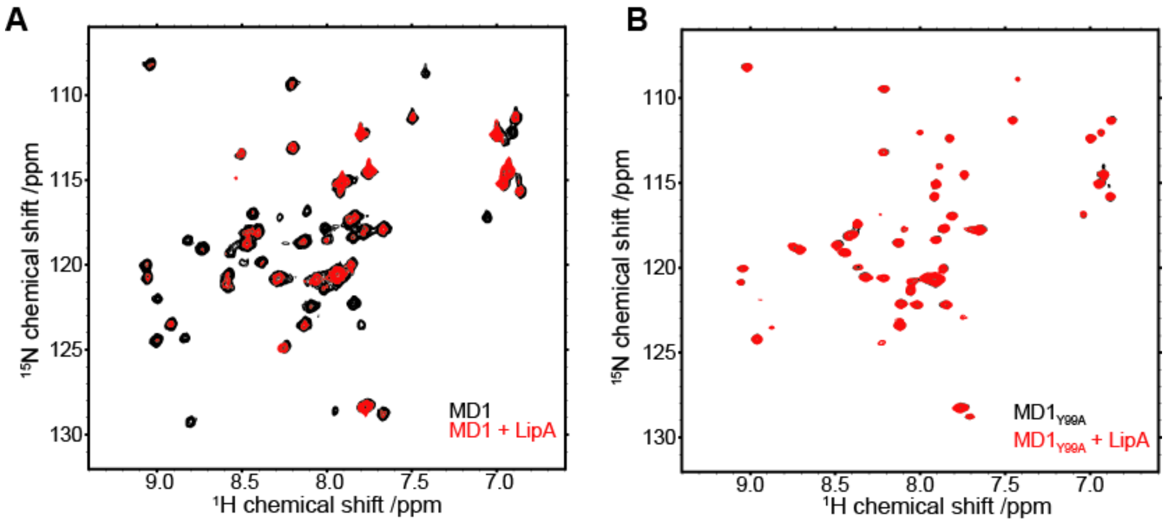

Figure S8 ^{1}H-^{15}N HSQC spectra of isotope labelled variants of MD1 in the absence (black) and presence of 3-fold molar excess of unlabeled LipA. (A and B) ^{1}H,^{15}N-HSQC spectra of MD1 and MD1$_{Y99A}$, respectively, in the presence (black) and absence of 3-fold molar excess of LipA (red). Note that the observed decrease in NMR signal intensity upon MD1-LipA interaction indicates binding in the NMR intermediate to slow exchange regime and that less effected peaks originate from nuclei located in the amino acids side chains or the protein's flexible regions. The relaxation properties of these positions should be less affected by the expected change in particle size upon interaction with the LipA. Overall, our data, therefore, do not allow to map the binding interface reliably.

Supplementary Tables

Table S1 Steady-state fluorescence anisotropy $r_{steady\text{-}state}$ and associated fraction of complex $x_{complex}$ of sLif labelled with BDP FL NHS ester with LipA at different concentrations of LipA.

LipA [μM]	r_{ss}	$x_{complex}$	LipA [μM]	r_{ss}	$x_{complex}$
0.000	0.101	0.084	0.002	0.108	0.348
0.000	0.097	-0.023	0.002	0.105	0.252
0.000	0.096	-0.062	0.005	0.107	0.329
0.001	0.105	0.257	0.015	0.106	0.272
0.002	0.098	0.011	0.044	0.116	0.630
0.004	0.117	0.666	0.13	0.121	0.818
0.008	0.097	-0.048	0.40	0.129	1.085
0.016	0.107	0.309	1.20	0.120	0.756
0.032	0.105	0.245	3.59	0.126	0.996
0.063	0.114	0.542	0.002	0.106	0.279
0.13	0.115	0.588	0.005	0.105	0.259
0.25	0.123	0.860	0.015	0.103	0.171
0.50	0.128	1.039	0.044	0.113	0.530
1.01	0.122	0.855	0.13	0.120	0.785
2.02	0.133	1.228	0.40	0.124	0.919
4.04	0.122	0.829	1.20	0.126	0.984
8.08	0.126	0.996	3.59	0.134	1.272
0.002	0.100	0.054	10.8	0.122	0.839

Table S2 Average translational diffusion time ‹t_{trans}› (eq. 3) and associated fraction of complex $x_{complex}$ of sLif$_{Y99A}$ labelled with BDP FL NHS ester with LipA at different concentrations of LipA.

LipA [µM]	‹t_{trans}› [ms]	$x_{complex}$	LipA [µM]	‹t_{trans}› [ms]	$x_{complex}$
0.0016	0.75	0.219	1.2	0.92	1.058
0.0016	0.73	0.102	3.6	0.84	0.678
0.0049	0.72	0.097	10.8	0.93	1.115
0.015	0.65	-0.290	32.3	0.87	0.832
0.044	0.81	0.542	0.0005	0.72	0.051
0.13	0.80	0.491	0.0005	0.74	0.158
0.40	0.95	1.227	0.0005	0.71	0.006
1.2	0.91	1.032	0.0010	0.66	-0.228
3.6	0.88	0.890	0.0020	0.72	0.082
10.8	0.91	1.029	0.0039	0.66	-0.218
32.3	0.91	1.041	0.0079	0.66	-0.222
0.0016	0.75	0.212	0.016	0.77	0.315
0.0049	0.77	0.330	0.032	0.69	-0.082
0.015	0.70	-0.048	0.063	0.83	0.598
0.044	0.82	0.583	0.126	0.85	0.706
0.13	0.79	0.427	0.252	0.80	0.474
0.40	0.86	0.766	0.505	0.89	0.899

Table S3 Fluorescence anisotropy decay $r(t_c)$ fit parameters from the model function given by eq. S4b, of MD1 and $MD1_{Y99A}$ labelled with BDP FL NHS ester, with different concentrations of unlabelled $MD1/MD1_{Y99A}$ and LipA. Condition #1 - 0.1 µM $MD1/MD1_{Y99A}$. Condition #2 - 0.1 mM $MD1/MD1_{Y99A}$ (NMR conditions). Condition #3 - 0.1 mM $MD1/MD1_{Y99A}$ and 0.4 mM LipA (NMR conditions). MD1 and $MD1_{Y99A}$ show similar hydrodynamic properties and both experience slower rotation at 0.1 mM concentrations (condition #2), which canbe explained by increased viscosity (factor 1.2 compared to condition #1). Assuming global rotational correlation time of MD1:LipA complex of ρ_{global} = 32 ns, a fit of fluorescence anisotropy decay requires approx. 20% of such complex fraction in case of both MD1 and $MD1_{Y99A}$ in presence of 0.4 mM LipA (condition #3). Fits are shown in Fig. S5.

	Condition	r_1	ρ_1 [ns]	r_2	ρ_2 [ns]	r_3	ρ_3 [ns]	r_4	ρ_4 [ns]	r_{ss}	$\chi_r 2$
**MD1**	#1					0.178	7.8[a]	0	0	0.162	
	#2	0.04	0.32	0.11	2.87	0.178	9.2	0	0	0.170	1.007
	#3					0.158	9.5[b]	0.024	32[c]	0.177	
**$MD1_{Y99}$**	#1					0.102	7.8[a]	0	0	0.129	
	#2	0.06	0.29	0.15	2.84	0.102	9.5	0	0	0.136	1.013
	#3					0.072	9.5[b]	0.039	32[c]	0.147	

[a]Global rotational correlation time estimated with HYDROPRO software [1] for $MD1/MD1_{Y99A}$ PDB code 5OVM at 20°C (viscosity 1.002 mPa·s).

[b]Global rotational correlation time of $MD1/MD1_{Y99A}$ in presence of 0.1 mM unlabelled $MD1/MD1_{Y99A}$ (NMR condition). Global rotation is approx. factor 1.2 slower, compared to sub-micromolar concentrations used for fluorescence measuremnets in condition #1, because of larger viscosity

[c]Global rotational correlation time of $MD1/MD1_{Y99A}$ in hypothetical complex with LipA estimated with HYDROPRO software [1] for PDB code 2ES4 with *bg*Lif replaced with MD1 (PDB code 5OVM, 20°C, 1.002 mPa·s, ρ_{global} = 26 ns) multiplied by factor 1.2.

Table S4 Fluorescence anisotropy decay $r(t_c)$ fit parameters.

A. sLif and $sLif_{Y99A}$ labelled with BDP FL NHS ester, in absence and presence of 40 μM LipA. Fluorescence decay $F(t_c)$ model parameters from the model function given by eq. S4a.

	x_1	τ_1 [ns]	x_2	τ_2 [ns]	x_3	τ_3 [ns]	a_3	τ_4 [ns]	$\langle\tau\rangle_x$ [ns]	$\langle\tau\rangle_F$ [ns]
sLif	0.471	6.03	0.390	3.67	0.090	1.16	0.048	0.249	4.56	5.27
+ LipA (40 μM)	0.600	5.89	0.277	3.43	0.76	0.78	0.046	0.130	4.55	5.30
$sLif_{Y99A}$	0.593	6.02	0.300	3.80	0.060	1.28	0.046	0.387	4.81	5.40
+ LipA (40 μM)	0.514	5.92	0.365	3.80	0.072	1.29	0.049	0.279	4.54	5.16

B. sLif and $sLif_{Y99A}$ labelled with BDP FL NHS ester, in absence and presence of 40 μM mM LipA. Fluorescence anisotropy decay $r(t_c)$ model parameters from the model function given by eq. S4b. Amplitude of global rotational correlation time (r_3) is similar for sLif and sLif:LipA complex, while global rotational correlation time ρ_3 changest from approx. 33 ns to 50 ns. In case of $sLif_{Y99A}$ ρ_3 shows similar behaviour but amplitude of global rotation (r_3) decreases significantly from 0.10 to 0.06. Fits are shown in **Figure** 6.

	r_1	ρ_1 [ns]	r_2	ρ_2 [ns]	r_3	ρ_3 [ns]	r_{ss}	$\chi_r 2$
sLif	0.13	0.21	0.09	1.72	0.12	33	0.129	1.017
+ LipA (40 μM)	0.12	0.20	0.07	1.48	0.14	50	0.148	1.008
$sLif_{Y99A}$	0.13	0.22	0.09	1.89	0.10	33	0.116	1.085
+ LipA (40 μM)	0.15	0.20	0.09	1.42	0.08	50	0.101	1.028

Table S5 pFCS fit parameters for *s*Lif and *s*Lif$_{Y99A}$ labeled with BDP FL from the model function $G(t_c)$ given by eq. S2, common fit parameters for 3 samples indicate a global optimization of these parameters in a free fit. Fits are shown in Fig. 3D.

**Parameters**	sLif	sLif:LipA	sLif$_{Y99A}$:LipA
		$G_{p1,p2}(t_c)$	
$\chi 2$	5.86	24.5	7.04
N	1.55±0.01	1.37±0.01	1.08±.01
t_{trans} [μs]	360±8	387±9	351±8
(z_0/w_0)		10.7	
b_1		0.155±0.007	
t_{b1} [μs]		136±11	
b_2		0.156±0.006	
t_{b2} [μs]		17.4±1.5	
b_3		0.127±.006	
t_{b3} [μs]		1.85±0.18	
a	0.923±0.014	0.913±0.011	0.910±0.010
t_a [ns]		4.37±0.14	
b_{rot}	0.519±0.036	0.640±0.024	0.514±0.020
ρ_{global} [ns]	**32±3**	**50±3**	**53±3**
$C^{\#}$		0.0889	
$S^{\#}$		0.3	

\# fixed spherical rotator parameters, Kask *et al.* [2]

Table S6 List of used bacterial strains, plasmids and oligonucleotides.

Strain	Genotype	Reference
E. coli DH5α	F– Φ80**lac**ZΔM15 Δ(**lac**ZYA-**arg**F) U169 **rec**A1 **end**A1 **hsd**R17 (rK–, mK+) **pho**A **sup**E44 λ– **thi**-1 **gyr**A96 **rel**A1	Hanahan (1983) [3]
E. coli BL21 (DE3)	*E. coli* B dcm ompT hsdS($r_B^- m_B^-$) gal, λDE3	Studier *et al.*(1986) [4]

Plasmid	Features	Reference
pLipA-SS	pET22b (NdeI, BamHI, 916 bp; *lipA Δ1-78 bp* coding for signal sequence, Ser1Met, under P_{T7} control), Amp^r	Hausmann *et al.* (2008) [5]
pEHTHis19	pET19b (NdeI, BamHI, 985 bp; lipH Δ1-60bp coding for TMD, substitution 84 bp His_{10}-tag and soluble linker, under P_{T7} control), Amp^r	Hausmann *et al.* (2008) [5]
pET-MD1	pET19b carrying the gene encoding MGH_6 sequence in the front of MD1 (amino acids 66-146)	this work
pET-MD1$_{Y99A}$	Y99A mutation inserted into pET-MD1 plasmid	this work
pET-*s*Lif$_{Y99A}$	Y99A mutation inserted into pEHTHis19 plasmid	this work

Oligonucleotide	Sequence
Lif_dLinkVD_fw	GGCCATCACCATCACCATCACCTGCCAACCTCCTTCAGGG
Lif_dLinkVD_rv	CAGGTGATGGTGATGGTGATGGCCCATGGTATATCTCC
Lif_Y99A_fw	ACATCCGCAACCTGTTCGACGCCTTCCTCAGCGCCGTCGGCG
LipH_Y99_rv	GTCGAACAGGTTGCGGATGTCGC
LipH_backbone_fw	TGACCGGCACGGAAACGC
LipH_MD1_rv	TTTCCGTGCCGGTCACAGTTCCTTCTTGTAGTCGATG

Table S7 Acquisition parameters of the spectra used for MD1 resonance assignment and structure calculation.

	Number of points			Spectral width (ppm)			Central frequency (ppm)			NS
Backbone assignment	F3	F2	F1	F3	F2	F1	F3	F2	F1	
2D										
$^{1}H,^{15}N$-HSQC	-	2048	256	-	12	30	-	4.695	119.5	64
$^{1}H,^{13}C$-HSQC	-	2048	512	-	12	75	-	4.695	39	64
3D										
HNCO	2048	40	128	12	36	22	4.706	119.5	176	8
HN(CA)CO	2048	40	128	12	36	22	4.706	119.5	176	16
HN(CO)CACB	2048	40	128	12	36	75	4.706	119.5	39	16
HNCACB*	2048	40	128	12	30	75	4.695	119.5	39	128
Side chain assignment										
(H)CCH-TOCSY	2048	64	128	12	75	75	4.706	39	39	16
NOE measurement										
3D- $^{1}H,^{15}N$-NOESY-HSQC (mix = 120 ms)	2048	40	120	12	30	12	4.695	119.5	4.695	32
3D- $^{1}H,^{13}C$-NOESY-HSQC (mix = 200 ms)	2048	40	120	12	75	12	4.695	39	4.695	32

* This experiment was recorded using non-uniformly sampling (NUS) of NMR data with 20 % sampling density. The NUS schedule was generated with the Poisson gap sampling method and the spectrum was subsequently reconstructed using hmsIST [6, 7] and processed with NMRpipe [8]). All other spectra were processed with Topspin3.5 (Bruker BioSpin).

Table S8 Acquisition parameters of the spectra used for MD1$_{Y99A}$ resonance assignment and structure calculation.

	Number of points			Spectral width (ppm)			Central frequency (ppm)			NS
Backbone assignment	F3	F2	F1	F3	F2	F1	F3	F2	F1	
2D										
^{1}H,^{15}N-HSQC	-	2048	256	-	12	30	-	4.695	119.5	64
^{1}H,^{13}C-HSQC	-	2048	512	-	12	75	-	4.695	39	64
3D										
HN(CA)CO	2048	40	128	12	30	22	4.702	119.5	176	8
HNCACB	2048	40	128	12	30	75	4.697	119.5	39	16
Side chain assignment										
(H)CCH-TOCSY	2048	56	120	12	75	75	4.697	39	39	16
NOE measurement										
3D- ^{1}H,^{15}N-NOESY-HSQC (mix = 250 ms)	2048	40	128	12	35	12	4.702	119.5	4.702	32
3D- ^{1}H,^{15}N-NOESY-HSQC (mix = 120 ms)	2048	40	128	12	30	12	4.695	119.5	4.695	28
3D- ^{1}H,^{13}C-NOESY-HSQC (mix = 200 ms)	2048	40	128	12	75	12	4.695	39	4.695	28

Supplementary Methods

Section S1. pFCS analysis

Polarization-resolved fluorescence correlation spectroscopy (pFCS), in which fluorescence intensity fluctuations under constant excitation are measured, is also able to resolve molecular rotational motion. Auto- and cross-correlations between different polarization channels were obtained were obtained according to Felekyan *et al.* [9]

$$G_{s1,s2}(t_c) = 1 + \frac{\langle \delta F_{s1}(t)\delta F_{s2}(t+t_c)\rangle}{\langle F_{s1}(t)\rangle\langle F_{s2}(t)\rangle}$$

$$G_{p1,p2}(t_c) = 1 + \frac{\langle \delta F_{p1}(t)\delta F_{p2}(t+t_c)\rangle}{\langle F_{p1}(t)\rangle\langle F_{p2}(t)\rangle}$$

$$G_{s,p}(t_c) = 1 + \frac{\langle \delta F_{s}(t)\delta F_{p}(t+t_c)\rangle}{\langle F_{s}(t)\rangle\langle F_{p}(t)\rangle}$$

$$G_{p,s}(t_c) = 1 + \frac{\langle \delta F_{p}(t)\delta F_{s}(t+t_c)\rangle}{\langle F_{s}(t)\rangle\langle F_{p}(t)\rangle} \qquad \text{(S1)}$$

with the fluorescence fluctuations, $\delta F(t) = F(t) - \langle F(t)\rangle$. The registered photon events were analyzed by employing a custom designed software package for multiparameter fluorescence spectroscopy, full correlation and multiparameter fluorescence imaging [9]. The applied factorized fitting function (eq S4) models translational diffusion in a 3D-Gaussian volume element $G_{trans}(t_c)$, up to three temporary dark states $G_b(t_c)$, rotational diffusion of a spherical rotator $G_{rot}(t_c)$ and photon antibunching $G_a(t_c)$:

$$G(t_c) = 1 + \frac{1}{N} G_a(t_c) \times G_b(t_c) \times G_{rot}(t_c) \times G_{trans}(t_c) \qquad \text{(S2)}$$

with

$$G_{trans}(t_c) = \left(1 + \frac{t_c}{t_{trans}}\right)^{-1}\left(1 + \left(\frac{\omega_0}{z_0}\right)^2 \times \frac{t_c}{t_{trans}}\right)^{-\frac{1}{2}} \qquad \text{(S2a)}$$

$$G_b(t_c) = 1 - b_1 + b_1 e^{-t_c/t_{b1}} - b_2 + b_2 e^{-t_c/t_{b2}} - b_3 + b_3 e^{-t_c/t_{b3}} \qquad \text{(S2b)}$$

$$G_a(t_c) = 1 - a e^{-t_c/t_a} \qquad \text{(S2c)}$$

$$G_{rot}(t_c) = 1 + b_{rot}\left(\frac{1}{1+C} e^{-t_c/\rho_{global}} + \frac{C}{1+C} e^{-t_c/\left(S\rho_{global}\right)}\right) \qquad \text{(S2d)}$$

Here, the observation volume is approximated by a 3D-Gaussian volume with $1/e^2$ radii in the lateral (ω_0) and axial direction (z_0), t_{trans} is the diffusion time, $b_{1,2,3}$ and $t_{b1,b2,b3}$ are amplitudes and times of the bunching terms, N is the particle number, a and t_a are the amplitude and time of the antibunching term, S and C characterize the rotation model (see Kask *et al.* [2]), b_{rot} and ρ_{global} are the amplitude and correlation time associated with rotational motion. As described above, factorization of the model function (eq S4) is based on the assumption of well-separated time scales for antibunching ($t_a \approx \tau_e$) and rotational correlation (ρ_{global}).

Section S2. Fluorescence anisotropy decay analysis

Polarization resolved fluorescence intensity decay histograms $F_p(t_c)$ and $F_s(t_c)$ were recorded. The fluorescence $F(t_c)$ and anisotropy decay $r(t_c)$ parameters were recovered by global fitting o the sum $F_{sum}(t_c)$ and difference $F_{dif}(t_c)$ histograms as previously described by Mockel *et al.* [10]

$$F_{sum}(t_c) = \left(F_p(t_c) + F_s(t_c)\right) = F(t_c) \quad \text{(S3a)}$$

$$F_{dif}(t_c) = \left(F_p(t_c) - F_s(t_c)\right) = F(t_c) \cdot r(t_c) \quad \text{(S3b)}$$

where fluorescence and anisotropy decays were modelled by a weighted sum of exponentials,

$$F(t_c) = \sum_{i=1}^{4} x_i \cdot \tau_i \text{ where } \sum x_i = 1 \quad \text{(S4a)}$$

and $r(t_c) = \sum_{i=1}^{4} r_i \cdot \rho_i$ (S4b)

with fluorescence lifetimes τ_i, their species fractions x_i, and with rotational correlation times ρ_i and their related amplitudes r_i. We checked that $\sum_{i=1}^{4} r_i \leq r_0$ with the fundamental anisotropy of BDP FL r_0=0.375.[11]

Order parameters of global rotational correlation time[12] were calculated according to [10]: $S^2 = r_3/r_0$.

Section S3. Determination of half-inactivation temperature.

Half-inactivation temperature T_{50}, i.e. the temperature at which LipA activity is reduced to 50% in a temperature-dependent lipase activity of *s*Lif and LipA generated by incubation of pre-active LipA (100 nM) with *s*Lif (250 nM) overnight at 4°C in TG buffer. Samples were then incubated at different temperatures (10 – 50°C) for 1 h followed by measurement of the remaining lipase activity with 2 nM LipA by *p*NPP-based lipase activity assay. Data were fitted according to:

$$A(T) = \frac{A_{max} - A_{min}}{1 + e^{(T - T_{50}/\partial T)}} + A_{min} \quad \text{(S5)}$$

where $A(T)$ is lipase activity at temperature T, A_{max} is maximum enzyme activity (here 100%) and A_{min} is minimum enzyme activity (here 0%), δT is a rate of activity loss. Half-inactivation temperature T_{50} was determined to 29.0 ± 0.2°C and 2.1 ± 0.2°C^{-1}.

Supplementary References

1. Ortega, A., Amoros, D., and de la Torre, J. G. (2011) Prediction of hydrodynamic and other solution properties of rigid proteins from atomic- and residue-level models. *Biophys J* **101**, 892-898
2. Kask, P., Piksarv, P., Pooga, M., Mets, U., and Lippmaa, E. (1989) Separation of the rotational contribution in fluorescence correlation experiments. *Biophys J* **55**, 213-220
3. Hanahan, D. (1983) Studies on transformation of *Escherichia coli* with plasmids. *Journal of Molecular Biology* **166**, 557-580
4. Studier, F. W., and Moffatt, B. A. (1986) Use of bacteriophage-T7 RNA-polymerase to direct selective high-level expression of cloned genes. *Journal of Molecular Biology* **189**, 113-130
5. Hausmann, S., Wilhelm, S., Jaeger, K. E., and Rosenau, F. (2008) Mutations towards enantioselectivity adversely affect secretion of *Pseudomonas aeruginosa* lipase. *FEMS Microbiol Lett* **282**, 65-72
6. Hyberts, S. G., Milbradt, A. G., Wagner, A. B., Arthanari, H., and Wagner, G. (2012) Application of iterative soft thresholding for fast reconstruction of NMR data non-uniformly sampled with multidimensional Poisson Gap scheduling. *Journal of Biomolecular Nmr* **52**, 315-327
7. Hyberts, S. G., Robson, S. A., and Wagner, G. (2013) Exploring signal-to-noise ratio and sensitivity in non-uniformly sampled multi-dimensional NMR spectra. *Journal of Biomolecular Nmr* **55**, 167-178
8. Delaglio, F., Grzesiek, S., Vuister, G. W., Zhu, G., Pfeifer, J., and Bax, A. (1995) Nmrpipe - a multidimensional spectral processing system based on unix pipes. *Journal of Biomolecular Nmr* **6**, 277-293
9. Felekyan, S., Kuhnemuth, R., Kudryavtsev, V., Sandhagen, C., Becker, W., and Seidel, C. A. M. (2005) Full correlation from picoseconds to seconds by time-resolved and time-correlated single photon detection. *Rev Sci Instrum* **76**
10. Mockel, C., Kubiak, J., Schillinger, O., Kuhnemuth, R., Della Corte, D., Schroder, G. F., Willbold, D., Strodel, B., Seidel, C. A. M., and Neudecker, P. (2019) Integrated NMR, fluorescence, and molecular dynamics benchmark study of protein mechanics and hydrodynamics. *J Phys Chem B* **123**, 1453-1480
11. Karolin, J., Johansson, L. B. A., Strandberg, L., and Ny, T. (1994) Fluorescence and absorption spectroscopic properties of dipyrrometheneboron difluoride (Bodipy) derivatives in liquids, Lipid-Membranes, and Proteins, *J Am Chem Soc 116*, 7801-7806.
12. Lipari, G., and Szabo, A. (1980) Effect of librational motion on fluorescence depolarization and nuclear magnetic-resonance relaxation in macromolecules and membranes, *Biophys J 30*, 489-506.
